# Differential Treatment Effects of Subgroup Analyses in Phase 3 Oncology Trials From 2004 to 2020

**DOI:** 10.1001/jamanetworkopen.2024.3379

**Published:** 2024-03-28

**Authors:** Alexander D. Sherry, Andrew W. Hahn, Zachary R. McCaw, Joseph Abi Jaoude, Ramez Kouzy, Timothy A. Lin, Bruce Minsky, C. David Fuller, Tomer Meirson, Pavlos Msaouel, Ethan B. Ludmir

**Affiliations:** 1 Department of Radiation Oncology, Division of Radiation Oncology, The University of Texas MD Anderson Cancer Center, Houston; 2 Department of Genitourinary Medical Oncology, Division of Cancer Medicine, The University of Texas MD Anderson Cancer Center, Houston; 3 Insitro, South San Francisco, San Francisco, California; 4 Department of Biostatistics, University of North Carolina at Chapel Hill; 5 Department of Radiation Oncology, Stanford University, Stanford, California; 6 Department of Radiation Oncology and Molecular Radiation Sciences, Johns Hopkins University School of Medicine, Baltimore, Maryland; 7 Department of Gastrointestinal Radiation Oncology, Division of Radiation Oncology, The University of Texas MD Anderson Cancer Center, Houston; 8 Davidoff Cancer Center, Rabin Medical Center, Petach Tikva, Israel; 9 Department of Translational Molecular Pathology, Division of Pathology/Lab Medicine, The University of Texas MD Anderson Cancer Center, Houston; 10 Department of Biostatistics, The University of Texas MD Anderson Cancer Center, Houston

## Abstract

**Importance:**

Subgroup analyses are often performed in oncology to investigate differential treatment effects and may even constitute the basis for regulatory approvals. Current understanding of the features, results, and quality of subgroup analyses is limited.

**Objective:**

To evaluate forest plot interpretability and credibility of differential treatment effect claims among oncology trials.

**Design, Setting, and Participants:**

This cross-sectional study included randomized phase 3 clinical oncology trials published prior to 2021. Trials were screened from ClinicalTrials.gov.

**Main Outcomes and Measures:**

Missing visual elements in forest plots were defined as a missing point estimate or use of a linear x-axis scale for hazard and odds ratios. Multiplicity of testing control was recorded. Differential treatment effect claims were rated using the Instrument for Assessing the Credibility of Effect Modification Analyses. Linear and logistic regressions evaluated associations with outcomes.

**Results:**

Among 785 trials, 379 studies (48%) enrolling 331 653 patients reported a subgroup analysis. The forest plots of 43% of trials (156 of 363) were missing visual elements impeding interpretability. While 4148 subgroup effects were evaluated, only 1 trial (0.3%) controlled for multiple testing. On average, trials that did not meet the primary end point conducted 2 more subgroup effect tests compared with trials meeting the primary end point (95% CI, 0.59-3.43 tests; *P* = .006). A total of 101 differential treatment effects were claimed across 15% of trials (55 of 379). Interaction testing was missing in 53% of trials (29 of 55) claiming differential treatment effects. Trials not meeting the primary end point were associated with greater odds of no interaction testing (odds ratio, 4.47; 95% CI, 1.42-15.55, *P* = .01). The credibility of differential treatment effect claims was rated as low or very low in 93% of cases (94 of 101).

**Conclusions and Relevance:**

In this cross-sectional study of phase 3 oncology trials, nearly half of trials presented a subgroup analysis in their primary publication. However, forest plots of these subgroup analyses largely lacked essential features for interpretation, and most differential treatment effect claims were not supported. Oncology subgroup analyses should be interpreted with caution, and improvements to the quality of subgroup analyses are needed.

## Key Points

**Question:**

Among phase 3 oncology trials, how often do subgroup analyses support claims of differential treatment effects?

**Findings:**

In this cross-sectional study of 379 published phase 3 randomized clinical trials with subgroup analyses, which enrolled 331 653 participants, most claims for differential treatment effects were rated as low or very low credibility according to the Instrument for Assessing the Credibility of Effect Modification Analyses.

**Meaning:**

In this study, the differential treatment effect claims of most phase 3 randomized clinical trials in oncology were not well-supported.

## Teaser

This cross-sectional study evaluates the credibility of differential treatment effect claims in published phase 3 randomized clinical oncology trials published prior to 2021 according to the Instrument for Assessing the Credibility of Effect Modification Analyses.

## Introduction

Phase 3 randomized clinical trials in oncology primarily compare the overall outcomes of 2 or more groups of patients.^1^ Acknowledging heterogeneity in enrolled patient characteristics, phase 3 trials often include additional comparisons to evaluate for differential treatment effects (DTEs) within patient subgroups.^2^ The results of such subgroup analyses suggesting DTEs have been used as the basis for regulatory approvals, even when the subgroup findings are in conflict with the overall trial results.^3^

However, despite their popularity and potential impact, subgroup analyses may be beset by multiple challenges.^4,5,6,7^ Phase 3 trials are usually not powered for subgroup analyses, which increases the risk of type II error; furthermore, repeated inferential testing examining multiple effects increases the risk of type I error.^8,9^ Forest plots presenting subgroup analyses at American Society of Clinical Oncology (ASCO) annual meetings appear to be uninformative and inconclusive in most cases.^10^ When DTEs are suspected based on visual inspection of the forest plot, interaction testing is recommended for further evaluation.^11,12,13,14,15^ However, interaction testing is often missing from subgroup analyses, and the alternative focus on *P* values within each subgroup is less informative and further inflates the risk of type I error.^6,16^ A landmark empirical evaluation of medical trials^17^ published in 2007 found that most claims of DTEs were not well-supported, highlighting the importance, problems, and consequences of DTE claims in subgroup analyses.

While multiple consensus statements offer guidelines on mitigating the challenges of subgroup analysis, the quality of modern published subgroup analyses is poorly understood.^13,18,19,20,21^ Because of the particular importance placed on subgroup analyses in oncology for regulatory approvals, a large-scale empirical evaluation of the published contemporary phase 3 oncology literature was undertaken to provide a current understanding of DTE claim credibility in oncology.^3^ The quality of DTE claims among phase 3 trials published between 2004 and 2020 was evaluated using the Instrument for Assessing the Credibility of Effect Modification Analyses (ICEMAN).^21^

## Methods

We performed a cross-sectional analysis of phase 3 randomized clinical trials in oncology. Trials were identified from the ClinicalTrials.gov registry without study date limitations using an advanced query through February 2020 with the search terms *cancer*; phase, *phase 3*; study results, *with results*; and status, *excluded: not yet recruiting*, as previously reported.^22^ The subgroup analyses of the article reporting the formal results of the primary end point for the trial were evaluated. This study adhered to the Strengthening the Reporting of Observational Studies in Epidemiology (STROBE) reporting guidelines.^23^ Per the Common Rule, institutional review board approval was not required as the data were publicly available.

**Figure 1.**
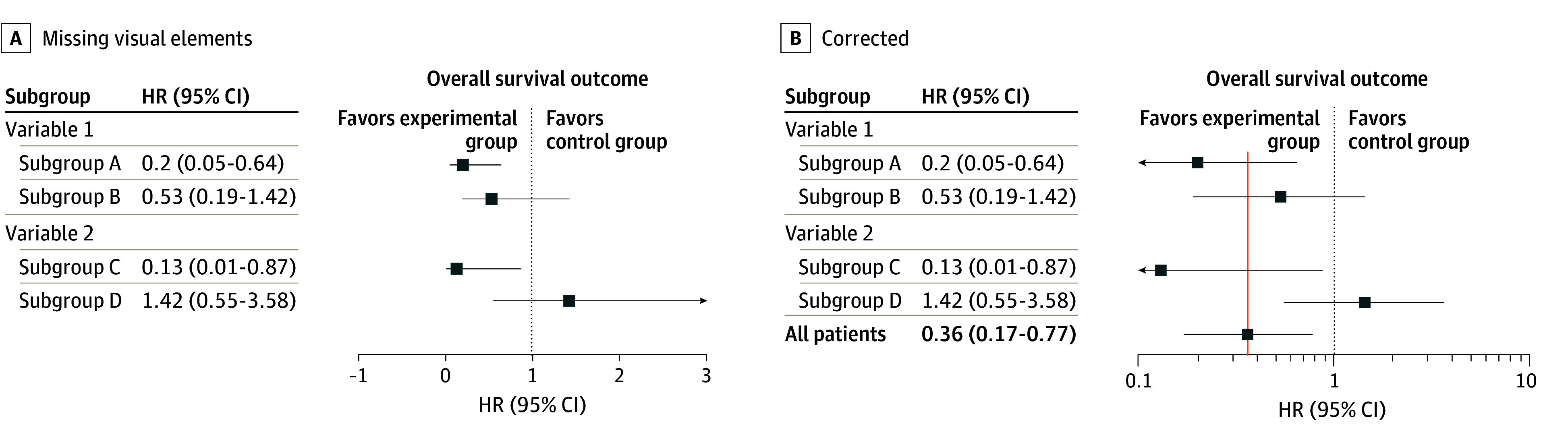
Illustrative Forest Plots With Missing Visual Elements and Corrected Figure represents a hypothetical randomized clinical trial comparing an experimental arm vs a control arm. HR indicates hazard ratio.

Multiplicity control in subgroup analyses by any established method was recorded, as larger numbers of subgroup analyses, even if prespecified, increase the risk of type I error.^13,19^ Credibility was rated for each DTE claim according to the ICEMAN guidelines.^21^ A DTE was defined as visually apparent based on crude inspection of the forest plot by the Cuzick method: if subgroup confidence intervals do not overlap with the point estimate of the main effect, DTEs may be present and merit further investigation (Figure 1).^10,24^ Forest plot missing visual elements (MVEs) were defined as either (1) utilization of a linear x-axis scale, as opposed to a logarithmic x-axis scale, for hazard ratios (HRs) or odds ratios (ORs) or (2) lack of a main effect present on the plot (Figure 1A).^10^ Plotting HRs dispersed linearly over the x-axis as in Figure 1A distorts the effect size appearance in favor of the control arm. Instead, HR values should be dispersed logarithmically in the x-axis space as in Figure 1B to preserve symmetry above and below 1. Furthermore, without the main effect present on the graph as shown in Figure 1A, interpretation of the subgroup analysis is substantially limited. To facilitate interpretation, it is recommended to include the main effect and to draw a line through the point estimate HRs of the main effect, so that overlap with subgroups can be easily visualized, as shown in Figure 1B. It is clear, in this example, that the 95% CI of subgroup D does not overlap with the point estimate of the main effect, suggesting potential DTEs by the Cuzick method. Although the 95% CI of subgroup B overlaps with 1, because the interval crosses the point estimate of the main effect, the visual interpretation for subgroup B suggests no evidence of DTEs. Subgroup B should also not be interpreted as lacking benefit of the experimental therapy. Finally, negative portions of the x-axis, as in Figure 1A, should not be present as negative HR are not possible.

Trial-level variables including eligibility for inclusion were manually curated into a standardized database with predefined variables by 4 trained reviewers (A.D.S., J.A.J., R.K., and T.A.L.) with data adjudication by the senior author (E.B.L.); MVEs and DTEs were evaluated by a single reviewer (A.D.S.).^25^ Trial-level variables included disease stage (ie, solid tumor metastatic, solid tumor nonmetastatic, or hematologic), disease site defined by the histology of the primary tumor, and the treatment being tested in the trial. Cooperative groups were defined as national or international nonprofit organizations typically receiving funding from governmental agencies (eg, the National Cancer Institute).^26^ Industry was defined as for-profit companies (pharmaceutical, biotechnology, and medical device companies). Because the statistical expertise available to industry trials may vary based on the size of the sponsor as well as other implications such as regulatory approvals, the industry sponsor was further subcharacterized as a larger vs smaller company.^27^ As there is no standardized definition, this was approximated by defining larger companies as those with an estimated annual revenue greater than US $1 billion, according to the most recent financial reports in the public domain. The primary end point of the trial was defined as met if the prespecified primary end point was statistically achieved in the article.^28^

### Statistical Analysis

**Figure 2.**
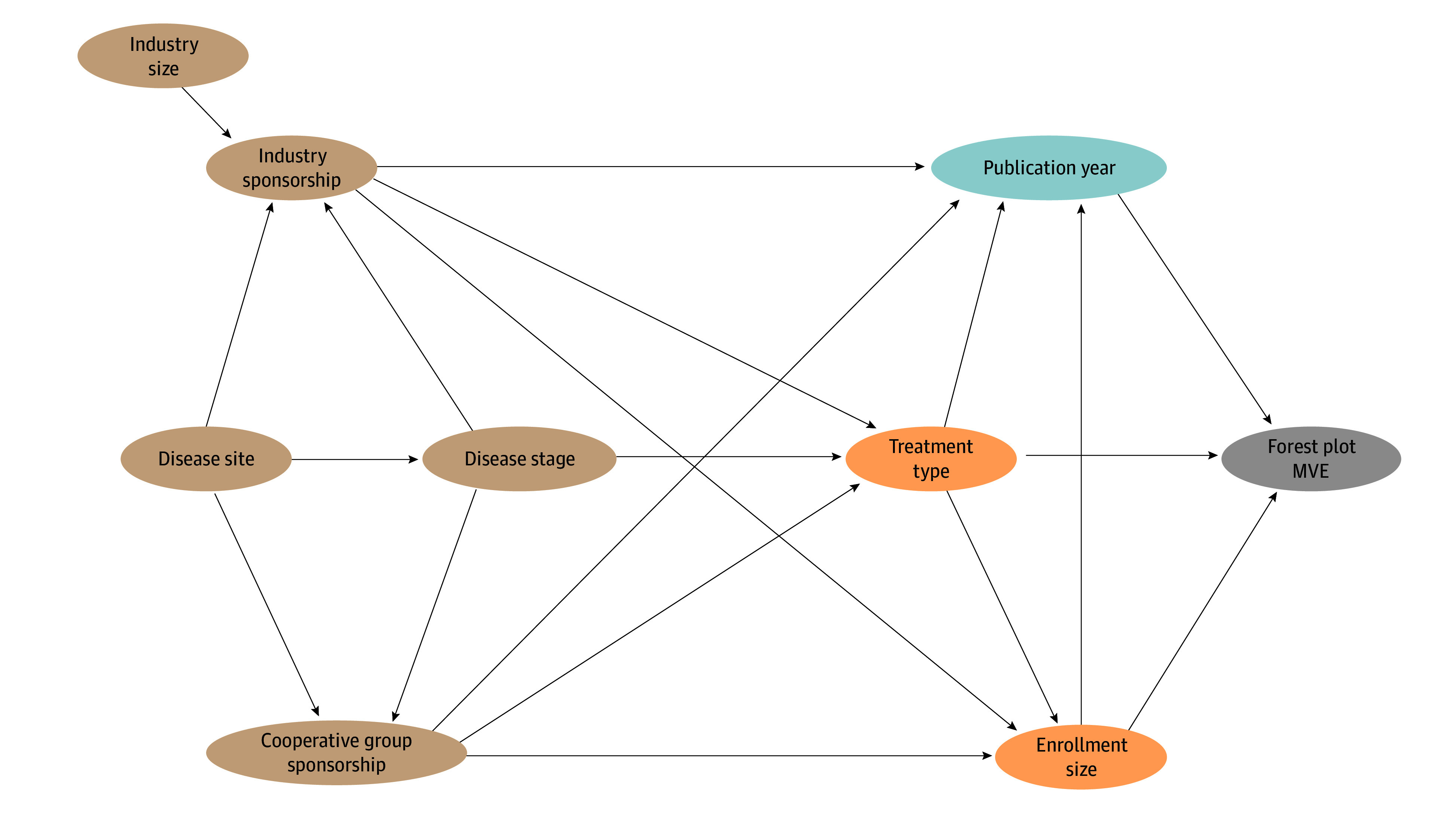
Directed Acyclic Graph for Publication Year, Other Variables, and Missing Visual Elements (MVEs) A light blue circle indicates the exposure of interest (publication year), and a gray circle represents the outcome of interest (MVE). Orange circles indicate confounders, and tan circles indicate nonconfounding ancestors of both the factor and outcome.

Descriptive statistics were used to summarize variables including frequency for categorical variables. Continuous variables were summarized by the median and IQR.^29^ Trends over time were examined by ordinary least squares linear regression, in which the slope (*m*) of the regression represented the rate of change. The association of trial-level variables and the number of subgroup tests was evaluated with linear regression. Univariable and multivariable logistic regressions were used to evaluate associations of trial-level factors and binary outcomes. Structural causal models were created for each factor on directed acyclic graphs using DAGitty to identify confounder variables (Figure 2).^30,31,32^ Effect directionality for each variable pair was drawn according to the most plausible causal pathway based on the investigators’ understanding of trial-level covariates. Each variable was sequentially selected as the factor of interest to identify confounder variables (eTable in Supplement 1). A multivariable logistic regression model for each variable, adjusting for confounders unique to that factor, examined the association of the variable of interest with MVEs using adjusted ORs (aORs). All tests were 2-sided. Significance (α) was set at .05, and confidence intervals were calculated at 95%. Missing data were not encountered. Analyses were performed using SAS version 9 (SAS Institute). The illustrative forest plots were created in R version 4.3.2 using forestploter (R Project for Statistical Computing). Other plots were created using Prism version 9 (GraphPad).

## Results

Based on a search of ClinicalTrials.gov, 785 published phase 3 interventional randomized trials were identified (eFigure in Supplement 1). Of these, 379 trials (48%) enrolling 331 653 patients reported the findings of a subgroup analysis and were eligible for study inclusion (eAppendix in Supplement 1). Trial publication dates ranged from 2004 to 2020.

The median number of subgroup factors analyzed per trial was 8 (IQR, 6-10; range, 1-29). A median of 1 outcome (eg, progression-free survival) was evaluated in subgroup analysis per trial (IQR, 1-2; range, 1-8). A total of 4148 subgroup effects were statistically evaluated, with a median of 9 subgroup effects per trial (IQR, 7-13; range, 1-58). One trial (0.3%) accounted for multiplicity of testing (using any method).^33^ On average, trials that did not meet the primary end point seemed to perform 2 more subgroup effect analyses than trials that met the primary end point (95% CI, 0.59-3.43 analyses; *P* = .006). Interaction testing was reported in 17% of trials (65 of 379).

Trial authors claimed DTEs in 15% of trials (55 of 379). Among the 55 trials claiming DTE, 101 total DTEs were claimed, appearing in the abstract in 29 instances (29%), in the results in all cases, in the discussion in 87 instances (86%), and in the conclusion in 39 cases (39%). DTE claims included the phrase “statistically significant… effect modification” in 4 cases (4%). ICEMAN ratings were mostly low credibility (69 [68%]) and very low credibility (25 [25%]), with only 7 DTE claims (7%) rated as moderately credible. Zero DTE claims were rated as highly credible. In only 5 of 101 instances (5%), interaction testing suggested that chance may not explain the apparent effect modification (interaction *P* value range, ≤.01 to >.005) or was an unlikely explanation (interaction *P* ≤ .005). In 78 DTE claims (77%), more than 10 effect modifiers were tested without regard to multiplicity (or the analysis was explicitly exploratory). Limited or indirect prior evidence, defined as retrospective evidence, nonsignificant effect modification in a prior randomized clinical trial, or a different patient population, supported the DTE claim in 40 cases (40%). Strong prior evidence, defined as a significant effect modification in a related randomized clinical trial, supported the DTE claim in 9 instances (9%). The direction of the effect modification was hypothesized a priori in 1 case (protocol was available) or probably hypothesized a priori in 6 cases (no protocol available).^34,35,36,37,38,39^ In only 2 cases, arbitrary cut points for continuous variables were definitely or probably avoided.^40,41^ Among trials claiming DTEs, only 27 had visually apparent DTEs suggested by the forest plot; moreover, the main effect was missing from the forest plot in 21% of these trials (11 of 52).

Interaction tests were missing in 53% (29 of 55) of trials claiming DTE. Unadjusted regression demonstrated that industry funded trials (OR, 6.00; 95% CI, 1.32-42.88; *P* = .03) were associated with higher odds of DTE claims lacking interaction testing; this association did not appear to be related to the size of the industry sponsor (larger vs smaller company: OR, 0.72; 95% CI, 0.09-4.16; *P* = .72). Trials that did not meet their primary end point were also more likely to claim DTE without interaction testing (OR, 4.47; 95% CI, 1.42-15.55, *P* = .01). There were no trial-level factors associated with greater credibility of DTE claims (moderate credibility vs low or very credibility, defined as the most credible DTE claim per trial).

**Figure 3.**
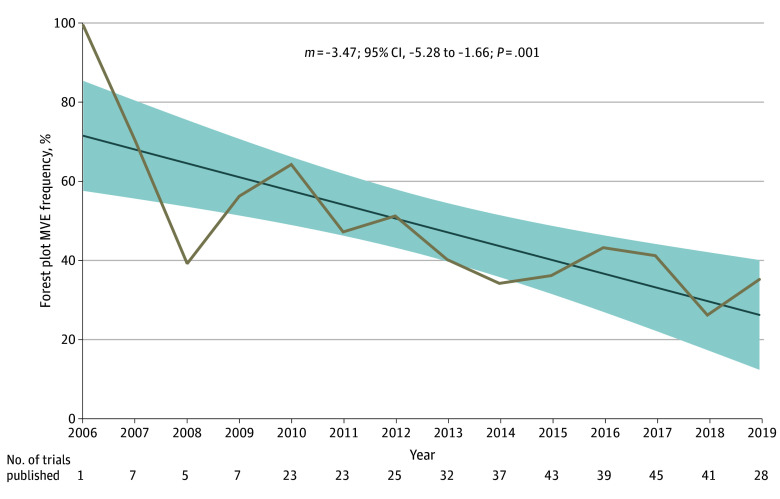
Trends in Forest Plot Missing Visual Elements (MVEs) The linear regression over time is shown. The shaded regions represent the 95% CI of the slope (*m*) of the linear regression. Because of the small number of trials analyzed in this dataset published in the years prior to 2006 (4 trials) or after 2019 (3 trials), data prior to 2006 or after 2019 were excluded from the graph.

Of 363 trials that included forest plots, 156 trials (43%) had forest plots with MVEs (Figure 1). Specifically, in 35% of trials (126 of 363), the x-axis scale representing HRs or ORs was linear rather than logarithmic. In 17% of trials (61 of 363), the main effect was not displayed in the forest plot. Over time, there appeared to be a significant decline in the rate of MVEs in forest plots (*m* = −3.47; 95% CI, −5.28 to −1.66; *P* = .001) (Figure 3). The rate of MVEs decreased from 62% (26 of 42 in 2007-2010) to 43% (50 of 117 in 2011-2014) and lastly to 37% (73 of 196 in 2015-2019).

Unadjusted associations between trial-level characteristics and forest plot MVEs are shown in Table 1, and adjusted associations are shown in Table 2. More recent publication year for the primary end point was associated with lower odds of MVE on multivariable analysis (publication year as a continuous factor: aOR, 0.89; 95% CI, 0.83-0.95; *P* < .001).

**Table 1.** Characteristics of Phase 3 Randomized Clinical Trials in Oncology and Forest Plot MVEs

Characteristic	Trials, No.	Forest Plot MVE, No. (%)		Unadjusted OR (95% CI)^a^	*P* value
		Yes	No		
Total trials	363	156 (43)	207 (57)	NA	NA
Disease stage					
Solid M0	67	31 (46)	36 (54)	1.05 (0.52-2.12)	.89
Solid M1	236	98 (42)	138 (58)	0.87 (0.49-1.54)	.63
Hematologic	60	27 (45)	33 (55)	1 [Reference]	NA
Disease site					
Breast	71	35 (49)	36 (51)	1.42 (0.71-2.87)	.33
Gastrointestinal	62	24 (39)	38 (61)	0.92 (0.44-1.91)	.82
Genitourinary	43	25 (56)	19 (44)	1.84 (0.84-4.12)	.13
Hematologic	60	27 (45)	33 (55)	1.19 (0.58-2.48)	.63
Thoracic	68	22 (32)	46 (68)	0.70 (0.34-1.44)	.33
Other^b^	59	24 (41)	35 (59)	1 [Reference]	NA
Treatment type					
Systemic therapy	350	151 (43)	199 (57)	1 [Reference]	NA
Supportive care	8	3 (38)	5 (62)	0.79 (0.16-3.27)	.75
Local therapy	5	2 (40)	3 (60)	0.88 (0.12-5.36)	.89
Cooperative study					
Yes	62	36 (58)	26 (42)	2.09 (1.20-3.67)	.009
No	301	120 (40)	181 (60)	1 [Reference]	NA
Industry funded					
Yes	328	134 (41)	194 (59)	0.41 (0.19-0.83)	.01
No	35	22 (63)	13 (37)	1 [Reference]	NA
Size of industry sponsor					
Larger	295	119 (40)	176 (60)	0.81 (0.39-1.69)	.57
Smaller	33	15 (45)	18 (55)	1 [Reference]	NA
Enrollment	NA	NA	NA	1.00 (1.00-1.00)	.49
Publication date^c^	NA	NA	NA	0.89 (0.83-0.95)	<.001

Abbreviations: M0, nonmetastatic; M1, metastatic; MVE, missing visual element; NA, not applicable; OR, odds ratio.

^a^ Associations were evaluated by univariable binary logistic regression.

^b^ Includes central nervous system, skin, endocrine, gynecologic, head and neck, and sarcoma.

^c^ Publication date is analyzed as a continuous variable, and the findings are shown in Figure 3.

**Table 2.** Multivariable Binary Logistic Regression Models Examining the Association of Trial-Level Factors With Missing Visual Elements in Forest Plots^a^

Characteristic	aOR (95% CI)	*P* value
Disease stage		
Solid M0	0.89 (0.38-2.08)	.79
Solid M1	0.82 (0.39-1.72)	.60
Hematologic	1 [Reference]	NA
Disease site^b^		
Breast	1.42 (0.71-2.87)	.33
Gastrointestinal	0.92 (0.44-1.91)	.82
Genitourinary	1.84 (0.84-4.12)	.13
Hematologic	1.19 (0.58-2.48)	.63
Thoracic	0.70 (0.34-1.44)	.33
Other^c^	1 [Reference]	NA
Treatment type		
Systemic therapy	1 [Reference]	NA
Supportive care	0.68 (0.13-2.90)	.61
Local therapy	0.40 (0.05-2.72)	.35
Cooperative study		
Yes	1.67 (0.81-3.47)	.16
No	1 [Reference]	NA
Industry funded		
Yes	0.60 (0.24-1.49)	.27
No	1 [Reference]	NA
Size of industry sponsor		
Larger	0.81 (0.39-1.69)	.57
Smaller	1 [Reference]	NA
Enrollment	1.00 (1.00-1.00)	.89
Publication date	0.89 (0.83-0.95)	<.001

Abbreviations: aOR, adjusted odds ratio; M0, non-metastatic; M1, metastatic; NA, not applicable.

^a^ Each trial-level factor was evaluated in a structural causal model depicted on a directed acyclic graph to identify confounders unique to its association with missing visual elements (see Figure 2 for an example). For each multivariable model, only the effect estimate of the primary factor of interest is reported. The effect estimates of confounders are not reported here, as they are not representative of their adjusted association with missing visual elements.

^b^ The structural causal model for disease site showed no confounding factors for the association with missing visual elements; therefore, the adjusted estimate for the association of disease site with missing visual elements is the same as the unadjusted model.

^c^ Includes central nervous system, skin, endocrine, gynecologic, head and neck, and sarcoma.

## Discussion

In this cross-sectional study, nearly half of phase 3 oncology trials presented the results of subgroup analyses in their primary article. Despite a large number of subgroup effects examined per trial, less than 1% of trials controlled for type I error rate. Forest plots largely lacked essential visual features needed for interpretation, and most claims for DTEs did not appear to be highly or moderately credible. Improvement to the conduct, quality, and interpretation of subgroup analyses in future phase 3 oncology trials is needed, and the subgroup analyses of current trials should be interpreted with caution.

This study has considerable implications for the interpretation of current phase 3 oncology research and future trials. Most claims of DTEs were scored as having low or very low credibility according to the ICEMAN guidelines.^21^ The consequences of DTE claims in prominent phase 3 trials can be considerable. In past cases, regulatory approvals have been granted to entire study populations based on positive subgroup findings, and in other cases, approvals have been limited just to subgroups despite positive results in the entire study population.^3^ Subgroup analyses may also drive the investment of substantial resources toward new trials, which may be particularly problematic if these are largely based on DTE claims with low credibility, in which there is increased risk of spurious results. Most subgroup analyses are at best hypothesis generating (except under highly specific conditions).^3,5,42,43^ An example is the ARTIST trial,^44^ which found no difference in disease-free survival between adjuvant chemotherapy and chemoradiotherapy for patients with gastric cancer with a D2 margin-negative resection but did suggest a possible benefit in patients with lymph node–positive disease based on a subgroup *P* value (without control for multiplicity of testing or use of interaction testing). The subsequent ARTIST 2 trial^45^ ultimately found no efficacy differences between chemoradiotherapy and chemotherapy among patients with lymph node–positive disease. This example, among others, illustrates why prudence and caution are needed in subgroup analysis interpretation. As suggested by the ICEMAN framework, interpretation of DTEs should be based on a variety of considerations, including strength of the interaction test, whether the directionality of the result was hypothesized a priori, the plausibility of the DTE in light of the underlying biology, the strength of the prior literature supporting the DTE, and the number of effects assessed, among other factors.^21^ Innovative approaches to subgroup analysis, such as using bayesian models or effect score analyses, may improve subgroup analyses in future phase 3 trials.^46,47,48^ For these and other reasons, subgroup analyses should not have a strong influence on overall trial interpretation.

While other studies have found that approximately one-quarter to one-half of clinical trials correct for multiple testing in some capacity (such as for the primary or secondary end points), the present study demonstrated a lack of this practice for subgroup analysis.^49,50,51^ Only 1 of 379 trials^33^ controlled for type I error in subgroup analyses, even though numerous effects were often analyzed per trial. An important caveat to this finding is that some trials may have conducted subgroup analyses to check for the overall consistency of effects within the sample rather than specifically evaluate for DTEs. Interestingly, in this study, trials that did not meet the primary end point seemed to conduct more subgroup effect tests than trials meeting the primary end point. Trialists should consider limiting the number of subgroup effects tested to those that are prespecified with directionality, biologically plausible, and supported by prior evidence, or if a larger number of effects are explored, controlling for type I error risk.^13,19,42,52,53,54,55^

While subgroup analyses were prevalent, most subgroup analyses in this study were not associated with DTE claims. This finding was similar to a prior study^10^ evaluating forest plots presented at ASCO annual meetings. Another notable observation of the current study was the discrepancy between authors’ claims of DTE and the use of interaction testing. This discrepancy appeared to be particularly evident in trials that did not meet their primary end point. Interaction testing is a crucial component of subgroup analysis when DTE is suspected and is preferred over subgroup *P* values.^4,5,9,12,13,56^ Two smaller studies^16,57^ similarly found low rates of interaction testing in subgroup analyses of metastatic solid tumor trials. Finally, accurate visual interpretation of subgroup analyses requires the presence of standard features in a forest plot.^10^ In this study, the forest plots of nearly half of trials lacked standard features needed for interpretation. The overall effect must be present on the graph to enable comparisons of the subgroups to the main effect for visual DTE interpretation (Figure 1). Furthermore, if a subgroup analysis entails ratios such as HRs or ORs, a logarithmic x-axis scale, rather than a linear x-axis scale, is needed to present effects symmetrically. Figure 1A shows a linear x-axis scale, and Figure 1B shows a corrected logarithmic scale. Encouragingly, the rate of MVEs seemed to decrease over time. This finding suggests the potential effectiveness of recent reporting guidelines, peer review, and editorial processes in mitigating visualization issues.^6,19,58^

### Limitations

There are several important limitations to this study. Trials were identified from ClinicalTrials.gov, and so our findings may not be generalizable to global trials. While ICEMAN offers a generalizable framework for evaluating DTE, ICEMAN credibility ratings were scored by a single reviewer. Multivariable adjustment was not performed in several analyses; their findings should be interpreted cautiously.

## Conclusions

In summary, despite finding that nearly half of phase 3 oncology trials use subgroup analyses, less than 1% of studies controlled for type I error rate, most claims of DTE do not appear to be supported, and many forest plots lack essential visual features. Critical improvements to the quality of subgroup analyses in future phase 3 oncology trials are necessary, and current subgroup analyses should be interpreted cautiously.

## Notes

Supplement 1. **eTable.** List of Confounders Identified for each Factor-Outcome Multivariable Regression **eFigure.** Trial Screening and Selection Diagram **eAppendix.** List of 378 References for 379 Trials Included in the Analysis

Supplement 2.
Data Sharing Statement
