# Supplementary material for: Differential Treatment Effects of Subgroup Analyses in Phase 3 Oncology Trials From 2004 to 2020
Source: JAMA Netw Open. 2024 Mar 28;7(3):e243379. doi: 10.1001/jamanetworkopen.2024.3379 (PMC10979321; doi:10.1001/jamanetworkopen.2024.3379)
Supplement: Supplement 1. — eTable. List of Confounders Identified for each Factor-Outcome Multivariable Regression eFigure. Trial Screening and Selection Diagram eAppendix. List of 378 References for 379 Trials Included in the Analysis [file jamanetwopen-e243379-s001.pdf]

Supplemental Online Content

Sherry AD, Hahn AW, McCaw ZR, et al. Differential treatment effects of subgroup analyses in phase 3 oncology trials from 2004 to 2020. *JAMA Netw Open.* 2024;7(3):e243379. doi:10.1001/jamanetworkopen.2024.3379

eTable. List of Confounders Identified for each Factor-Outcome Multivariable Regression

eFigure. Trial Screening and Selection Diagram

eAppendix. List of 378 References for 379 Trials Included in the Analysis

This supplemental material has been provided by the authors to give readers additional information about their work.

© 2024 Sherry AD et al. *JAMA Network Open.*

eTable. List of Confounders Identified for each Factor-Outcome Multivariable Regression

Confounders were identified by sequentially selected each variable as the predictor in the directed acyclic graph in **Figure 2**. The structural causal model for **Figure 2** was developed based on defining the most plausible causal pathways according to the investigators. The outcome of interest was the presence or absence of missing visual elements in the forest plot of the subgroup analysis.

**Predictor**	**Confounder(s)**
Enrollment	Cooperative group sponsorship, industry funding, treatment modality
Treatment modality	Cooperative group sponsorship and industry funding
Industry funding	Cooperative group sponsorship and disease stage
Size of Industry sponsor	None identified
Disease stage	Disease histology
Disease histology	None identified
Cooperative group sponsorship	Disease stage and industry funding
Year of publication	Treatment modality and enrollment

© 2024 Sherry AD et al. *JAMA Network Open.*

eFigure. Trial Screening and Selection Diagram

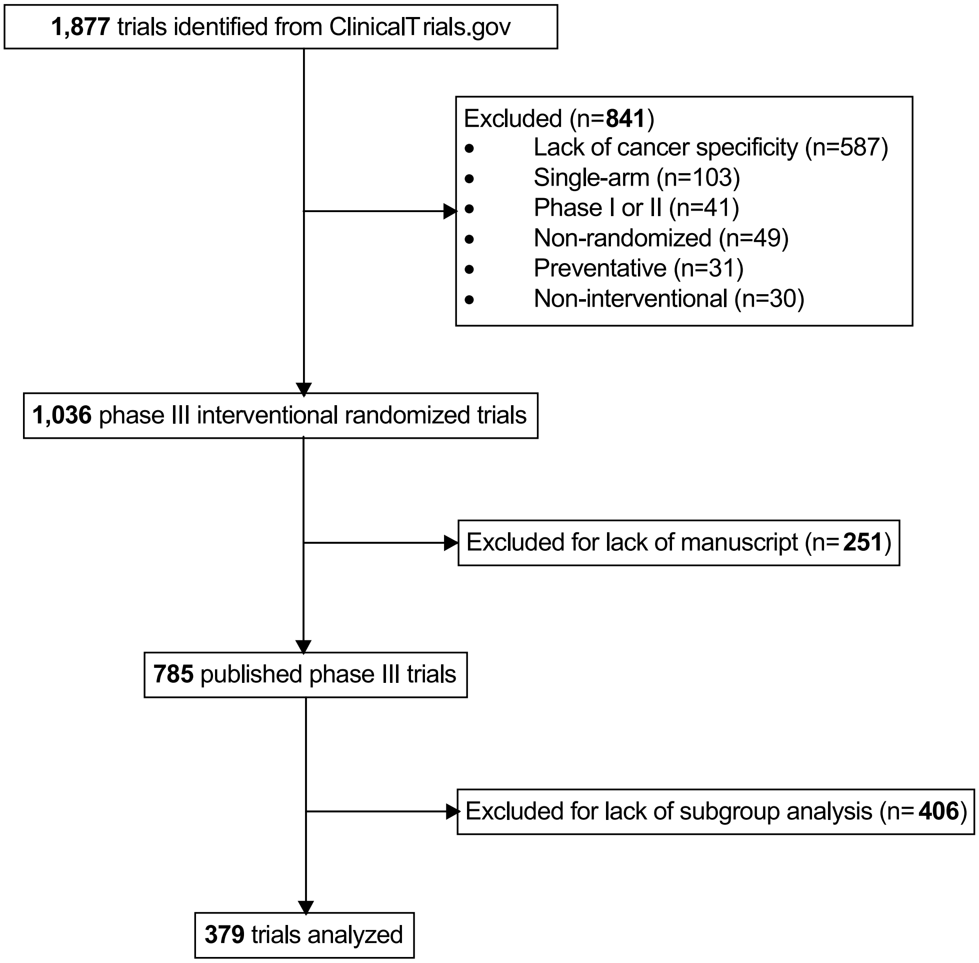

© 2024 Sherry AD et al. *JAMA Network Open.*

eAppendix. List of 378 References for 379 Trials Included in the Analysis.[1-378]

Of note, the primary endpoints of two trials were reported in one article.

1. Schuette W, Schneider CP, Engel-Riedel W, et al. 65Plus: open-label study of bevacizumab in combination with pemetrexed or pemetrexed/carboplatin as first-line treatment of patients with advanced or recurrent nonsquamous non-small-cell lung cancer. *Lung Cancer (Auckl)*. 2017;8:217-229. doi:10.2147/lctt.S142972

2. de Bono JS, Logothetis CJ, Molina A, et al. Abiraterone and increased survival in metastatic prostate cancer. *N Engl J Med*. May 26 2011;364(21):1995-2005. doi:10.1056/NEJMoa1014618

3. Ryan CJ, Smith MR, de Bono JS, et al. Abiraterone in metastatic prostate cancer without previous chemotherapy. *N Engl J Med*. Jan 10 2013;368(2):138-48. doi:10.1056/NEJMoa1209096

4. Livi L, Meattini I, Marrazzo L, et al. Accelerated partial breast irradiation using intensity-modulated radiotherapy versus whole breast irradiation: 5-year survival analysis of a phase 3 randomised controlled trial. *Eur J Cancer*. Mar 2015;51(4):451-463. doi:10.1016/j.ejca.2014.12.013

5. Van Cutsem E, Tabernero J, Lakomy R, et al. Addition of aflibercept to fluorouracil, leucovorin, and irinotecan improves survival in a phase III randomized trial in patients with metastatic colorectal cancer previously treated with an oxaliplatin-based regimen. *J Clin Oncol*. Oct 1 2012;30(28):3499-506. doi:10.1200/jco.2012.42.8201

6. Hawkins DS, Chi YY, Anderson JR, et al. Addition of Vincristine and Irinotecan to Vincristine, Dactinomycin, and Cyclophosphamide Does Not Improve Outcome for Intermediate-Risk Rhabdomyosarcoma: A Report From the Children's Oncology Group. *J Clin Oncol*. Sep 20 2018;36(27):2770-2777. doi:10.1200/jco.2018.77.9694

7. Cameron D, Brown J, Dent R, et al. Adjuvant bevacizumab-containing therapy in triple-negative breast cancer (BEATRICE): primary results of a randomised, phase 3 trial. *Lancet Oncol*. Sep 2013;14(10):933-42. doi:10.1016/s1470-2045(13)70335-8

8. Kang YK, Chang HM, Yook JH, et al. Adjuvant chemotherapy for gastric cancer: a randomised phase 3 trial of mitomycin-C plus either short-term doxifluridine or long-

term doxifluridine plus cisplatin after curative D2 gastrectomy (AMC0201). *Br J Cancer*. Apr 2 2013;108(6):1245-51. doi:10.1038/bjc.2013.86

9. Matei D, Filiaci V, Randall ME, et al. Adjuvant Chemotherapy plus Radiation for Locally Advanced Endometrial Cancer. *N Engl J Med*. Jun 13 2019;380(24):2317-2326. doi:10.1056/NEJMoa1813181

10. Wakelee HA, Dahlberg SE, Keller SM, et al. Adjuvant chemotherapy with or without bevacizumab in patients with resected non-small-cell lung cancer (E1505): an open-label, multicentre, randomised, phase 3 trial. *Lancet Oncol*. Dec 2017;18(12):1610-1623. doi:10.1016/s1470-2045(17)30691-5

11. Long GV, Hauschild A, Santinami M, et al. Adjuvant Dabrafenib plus Trametinib in Stage III BRAF-Mutated Melanoma. *N Engl J Med*. Nov 9 2017;377(19):1813-1823. doi:10.1056/NEJMoa1708539

12. Gnant M, Pfeiler G, Dubsky PC, et al. Adjuvant denosumab in breast cancer (ABCSG-18): a multicentre, randomised, double-blind, placebo-controlled trial. *Lancet*. Aug 1 2015;386(9992):433-43. doi:10.1016/s0140-6736(15)60995-3

13. Coleman R, Finkelstein DM, Barrios C, et al. Adjuvant denosumab in early breast cancer (D-CARE): an international, multicentre, randomised, controlled, phase 3 trial. *Lancet Oncol*. Jan 2020;21(1):60-72. doi:10.1016/s1470-2045(19)30687-4

14. Martin M, Pienkowski T, Mackey J, et al. Adjuvant docetaxel for node-positive breast cancer. *N Engl J Med*. Jun 2 2005;352(22):2302-13. doi:10.1056/NEJMoa043681

15. Witzig TE, Tobinai K, Rigacci L, et al. Adjuvant everolimus in high-risk diffuse large B-cell lymphoma: final results from the PILLAR-2 randomized phase III trial. *Ann Oncol*. Mar 1 2018;29(3):707-714. doi:10.1093/annonc/mdx764

16. Pagani O, Regan MM, Walley BA, et al. Adjuvant exemestane with ovarian suppression in premenopausal breast cancer. *N Engl J Med*. Jul 10 2014;371(2):107-18. doi:10.1056/NEJMoa1404037

17. Lee JH, Lee JH, Lim YS, et al. Adjuvant immunotherapy with autologous cytokine-induced killer cells for hepatocellular carcinoma. *Gastroenterology*. Jun 2015;148(7):1383-91.e6. doi:10.1053/j.gastro.2015.02.055

© 2024 Sherry AD et al. *JAMA Network Open.*

18. Eggermont AM, Chiarion-Sileni V, Grob JJ, et al. Adjuvant ipilimumab versus placebo after complete resection of high-risk stage III melanoma (EORTC 18071): a randomised, double-blind, phase 3 trial. *Lancet Oncol*. May 2015;16(5):522-30. doi:10.1016/s1470-2045(15)70122-1

19. Piccart-Gebhart M, Holmes E, Baselga J, et al. Adjuvant Lapatinib and Trastuzumab for Early Human Epidermal Growth Factor Receptor 2-Positive Breast Cancer: Results From the Randomized Phase III Adjuvant Lapatinib and/or Trastuzumab Treatment Optimization Trial. *J Clin Oncol*. Apr 1 2016;34(10):1034-42. doi:10.1200/jco.2015.62.1797

20. Goss PE, Smith IE, O'Shaughnessy J, et al. Adjuvant lapatinib for women with early-stage HER2-positive breast cancer: a randomised, controlled, phase 3 trial. *Lancet Oncol*. Jan 2013;14(1):88-96. doi:10.1016/s1470-2045(12)70508-9

21. Francis PA, Regan MM, Fleming GF, et al. Adjuvant ovarian suppression in premenopausal breast cancer. *N Engl J Med*. Jan 29 2015;372(5):436-46. doi:10.1056/NEJMoa1412379

22. Eggermont AMM, Blank CU, Mandala M, et al. Adjuvant Pembrolizumab versus Placebo in Resected Stage III Melanoma. *N Engl J Med*. May 10 2018;378(19):1789-1801. doi:10.1056/NEJMoa1802357

23. von Minckwitz G, Procter M, de Azambuja E, et al. Adjuvant Pertuzumab and Trastuzumab in Early HER2-Positive Breast Cancer. *N Engl J Med*. Jul 13 2017;377(2):122-131. doi:10.1056/NEJMoa1703643

24. Haas NB, Manola J, Uzzo RG, et al. Adjuvant sunitinib or sorafenib for high-risk, non-metastatic renal-cell carcinoma (ECOG-ACRIN E2805): a double-blind, placebo-controlled, randomised, phase 3 trial. *Lancet*. May 14 2016;387(10032):2008-16. doi:10.1016/s0140-6736(16)00559-6

25. van de Velde CJ, Rea D, Seynaeve C, et al. Adjuvant tamoxifen and exemestane in early breast cancer (TEAM): a randomised phase 3 trial. *Lancet*. Jan 22 2011;377(9762):321-31. doi:10.1016/s0140-6736(10)62312-4

26. Maio M, Lewis K, Demidov L, et al. Adjuvant vemurafenib in resected, BRAF(V600) mutation-positive melanoma (BRIM8): a randomised, double-blind, placebo-controlled, multicentre, phase 3 trial. *Lancet Oncol*. Apr 2018;19(4):510-520. doi:10.1016/s1470-2045(18)30106-2

© 2024 Sherry AD et al. *JAMA Network Open.*

27. Chamie K, Donin NM, Klöpfer P, et al. Adjuvant Weekly Girentuximab Following Nephrectomy for High-Risk Renal Cell Carcinoma: The ARISER Randomized Clinical Trial. *JAMA Oncol*. Jul 1 2017;3(7):913-920. doi:10.1001/jamaoncol.2016.4419

28. Harbeck N, Huang CS, Hurvitz S, et al. Afatinib plus vinorelbine versus trastuzumab plus vinorelbine in patients with HER2-overexpressing metastatic breast cancer who had progressed on one previous trastuzumab treatment (LUX-Breast 1): an open-label, randomised, phase 3 trial. *Lancet Oncol*. Mar 2016;17(3):357-366. doi:10.1016/s1470-2045(15)00540-9

29. Wu YL, Zhou C, Hu CP, et al. Afatinib versus cisplatin plus gemcitabine for first-line treatment of Asian patients with advanced non-small-cell lung cancer harbouring EGFR mutations (LUX-Lung 6): an open-label, randomised phase 3 trial. *Lancet Oncol*. Feb 2014;15(2):213-22. doi:10.1016/s1470-2045(13)70604-1

30. Soria JC, Felip E, Cobo M, et al. Afatinib versus erlotinib as second-line treatment of patients with advanced squamous cell carcinoma of the lung (LUX-Lung 8): an open-label randomised controlled phase 3 trial. *Lancet Oncol*. Aug 2015;16(8):897-907. doi:10.1016/s1470-2045(15)00006-6

31. Machiels JP, Haddad RI, Fayette J, et al. Afatinib versus methotrexate as second-line treatment in patients with recurrent or metastatic squamous-cell carcinoma of the head and neck progressing on or after platinum-based therapy (LUX-Head & Neck 1): an open-label, randomised phase 3 trial. *Lancet Oncol*. May 2015;16(5):583-94. doi:10.1016/s1470-2045(15)70124-5

32. Miller VA, Hirsh V, Cadranel J, et al. Afatinib versus placebo for patients with advanced, metastatic non-small-cell lung cancer after failure of erlotinib, gefitinib, or both, and one or two lines of chemotherapy (LUX-Lung 1): a phase 2b/3 randomised trial. *Lancet Oncol*. May 2012;13(5):528-38. doi:10.1016/s1470-2045(12)70087-6

33. Burtness B, Haddad R, Dinis J, et al. Afatinib vs Placebo as Adjuvant Therapy After Chemoradiotherapy in Squamous Cell Carcinoma of the Head and Neck: A Randomized Clinical Trial. *JAMA Oncol*. Aug 1 2019;5(8):1170-1180. doi:10.1001/jamaoncol.2019.1146

34. Li J, Xu R, Qin S, et al. Aflibercept plus FOLFIRI in Asian patients with pretreated metastatic colorectal cancer: a randomized Phase III study. *Future Oncol*. Aug 2018;14(20):2031-2044. doi:10.2217/fon-2017-0669

© 2024 Sherry AD et al. *JAMA Network Open.*

35. Novello S, Mazières J, Oh IJ, et al. Alectinib versus chemotherapy in crizotinib-pretreated anaplastic lymphoma kinase (ALK)-positive non-small-cell lung cancer: results from the phase III ALUR study. *Ann Oncol*. Jun 1 2018;29(6):1409-1416. doi:10.1093/annonc/mdy121

36. Hida T, Nokihara H, Kondo M, et al. Alectinib versus crizotinib in patients with ALK-positive non-small-cell lung cancer (J-ALEX): an open-label, randomised phase 3 trial. *Lancet*. Jul 1 2017;390(10089):29-39. doi:10.1016/s0140-6736(17)30565-2

37. Zhou C, Kim SW, Reungwetwattana T, et al. Alectinib versus crizotinib in untreated Asian patients with anaplastic lymphoma kinase-positive non-small-cell lung cancer (ALESIA): a randomised phase 3 study. *Lancet Respir Med*. May 2019;7(5):437-446. doi:10.1016/s2213-2600(19)30053-0

38. André F, Ciruelos E, Rubovszky G, et al. Alpelisib for PIK3CA-Mutated, Hormone Receptor-Positive Advanced Breast Cancer. *N Engl J Med*. May 16 2019;380(20):1929-1940. doi:10.1056/NEJMoa1813904

39. Parker C, Nilsson S, Heinrich D, et al. Alpha emitter radium-223 and survival in metastatic prostate cancer. *N Engl J Med*. Jul 18 2013;369(3):213-23. doi:10.1056/NEJMoa1213755

40. Temel JS, Abernethy AP, Currow DC, et al. Anamorelin in patients with non-small-cell lung cancer and cachexia (ROMANA 1 and ROMANA 2): results from two randomised, double-blind, phase 3 trials. *Lancet Oncol*. Apr 2016;17(4):519-531. doi:10.1016/s1470-2045(15)00558-6

41. Fernandez HF, Sun Z, Yao X, et al. Anthracycline dose intensification in acute myeloid leukemia. *N Engl J Med*. Sep 24 2009;361(13):1249-59. doi:10.1056/NEJMoa0904544

42. Smith MR, Saad F, Chowdhury S, et al. Apalutamide Treatment and Metastasis-free Survival in Prostate Cancer. *N Engl J Med*. Apr 12 2018;378(15):1408-1418. doi:10.1056/NEJMoa1715546

43. Armstrong AJ, Szmulewitz RZ, Petrylak DP, et al. ARCHES: A Randomized, Phase III Study of Androgen Deprivation Therapy With Enzalutamide or Placebo in Men With Metastatic Hormone-Sensitive Prostate Cancer. *J Clin Oncol*. Nov 10 2019;37(32):2974-2986. doi:10.1200/jco.19.00799

© 2024 Sherry AD et al. *JAMA Network Open.*

44. West H, McCleod M, Hussein M, et al. Atezolizumab in combination with carboplatin plus nab-paclitaxel chemotherapy compared with chemotherapy alone as first-line treatment for metastatic non-squamous non-small-cell lung cancer (IMpower130): a multicentre, randomised, open-label, phase 3 trial. *Lancet Oncol*. Jul 2019;20(7):924-937. doi:10.1016/s1470-2045(19)30167-6

45. Rini BI, Powles T, Atkins MB, et al. Atezolizumab plus bevacizumab versus sunitinib in patients with previously untreated metastatic renal cell carcinoma (IMmotion151): a multicentre, open-label, phase 3, randomised controlled trial. *Lancet*. Jun 15 2019;393(10189):2404-2415. doi:10.1016/s0140-6736(19)30723-8

46. Rittmeyer A, Barlesi F, Waterkamp D, et al. Atezolizumab versus docetaxel in patients with previously treated non-small-cell lung cancer (OAK): a phase 3, open-label, multicentre randomised controlled trial. *Lancet*. Jan 21 2017;389(10066):255-265. doi:10.1016/s0140-6736(16)32517-x

47. Eng C, Kim TW, Bendell J, et al. Atezolizumab with or without cobimetinib versus regorafenib in previously treated metastatic colorectal cancer (IMblaze370): a multicentre, open-label, phase 3, randomised, controlled trial. *Lancet Oncol*. Jun 2019;20(6):849-861. doi:10.1016/s1470-2045(19)30027-0

48. Johnson BE, Kabbinavar F, Fehrenbacher L, et al. ATLAS: randomized, double-blind, placebo-controlled, phase IIIB trial comparing bevacizumab therapy with or without erlotinib, after completion of chemotherapy, with bevacizumab for first-line treatment of advanced non-small-cell lung cancer. *J Clin Oncol*. Nov 1 2013;31(31):3926-34. doi:10.1200/jco.2012.47.3983

49. Leonard JP, Trneny M, Izutsu K, et al. AUGMENT: A Phase III Study of Lenalidomide Plus Rituximab Versus Placebo Plus Rituximab in Relapsed or Refractory Indolent Lymphoma. *J Clin Oncol*. May 10 2019;37(14):1188-1199. doi:10.1200/jco.19.00010

50. Stadtmauer EA, Pasquini MC, Blackwell B, et al. Autologous Transplantation, Consolidation, and Maintenance Therapy in Multiple Myeloma: Results of the BMT CTN 0702 Trial. *J Clin Oncol*. Mar 1 2019;37(7):589-597. doi:10.1200/jco.18.00685

51. Barlesi F, Vansteenkiste J, Spigel D, et al. Avelumab versus docetaxel in patients with platinum-treated advanced non-small-cell lung cancer (JAVELIN Lung 200): an open-label, randomised, phase 3 study. *Lancet Oncol*. Nov 2018;19(11):1468-1479. doi:10.1016/s1470-2045(18)30673-9

© 2024 Sherry AD et al. *JAMA Network Open.*

52. Gianni L, Romieu GH, Lichinitser M, et al. AVEREL: a randomized phase III Trial evaluating bevacizumab in combination with docetaxel and trastuzumab as first-line therapy for HER2-positive locally recurrent/metastatic breast cancer. *J Clin Oncol*. May 10 2013;31(14):1719-25. doi:10.1200/jco.2012.44.7912

53. Hutson TE, Lesovoy V, Al-Shukri S, et al. Axitinib versus sorafenib as first-line therapy in patients with metastatic renal-cell carcinoma: a randomised open-label phase 3 trial. *Lancet Oncol*. Dec 2013;14(13):1287-94. doi:10.1016/s1470-2045(13)70465-0

54. Ohtsu A, Shah MA, Van Cutsem E, et al. Bevacizumab in combination with chemotherapy as first-line therapy in advanced gastric cancer: a randomized, double-blind, placebo-controlled phase III study. *J Clin Oncol*. Oct 20 2011;29(30):3968-76. doi:10.1200/jco.2011.36.2236

55. Cunningham D, Lang I, Marcuello E, et al. Bevacizumab plus capecitabine versus capecitabine alone in elderly patients with previously untreated metastatic colorectal cancer (AVEX): an open-label, randomised phase 3 trial. *Lancet Oncol*. Oct 2013;14(11):1077-1085. doi:10.1016/s1470-2045(13)70154-2

56. von Minckwitz G, Puglisi F, Cortes J, et al. Bevacizumab plus chemotherapy versus chemotherapy alone as second-line treatment for patients with HER2-negative locally recurrent or metastatic breast cancer after first-line treatment with bevacizumab plus chemotherapy (TANIA): an open-label, randomised phase 3 trial. *Lancet Oncol*. Oct 2014;15(11):1269-78. doi:10.1016/s1470-2045(14)70439-5

57. Escudier B, Pluzanska A, Koralewski P, et al. Bevacizumab plus interferon alfa-2a for treatment of metastatic renal cell carcinoma: a randomised, double-blind phase III trial. *Lancet*. Dec 22 2007;370(9605):2103-11. doi:10.1016/s0140-6736(07)61904-7

58. Zielinski C, Láng I, Inbar M, et al. Bevacizumab plus paclitaxel versus bevacizumab plus capecitabine as first-line treatment for HER2-negative metastatic breast cancer (TURANDOT): primary endpoint results of a randomised, open-label, non-inferiority, phase 3 trial. *Lancet Oncol*. Sep 2016;17(9):1230-9. doi:10.1016/s1470-2045(16)30154-1

59. Miles D, Cameron D, Bondarenko I, et al. Bevacizumab plus paclitaxel versus placebo plus paclitaxel as first-line therapy for HER2-negative metastatic breast cancer (MERiDiAN): A double-blind placebo-controlled randomised phase III trial with prospective biomarker evaluation. *Eur J Cancer*. Jan 2017;70:146-155. doi:10.1016/j.ejca.2016.09.024

© 2024 Sherry AD et al. *JAMA Network Open.*

60. Chinot OL, Wick W, Mason W, et al. Bevacizumab plus radiotherapy-temozolomide for newly diagnosed glioblastoma. *N Engl J Med*. Feb 20 2014;370(8):709-22. doi:10.1056/NEJMoa1308345

61. Kantarjian H, Stein A, Gökbuget N, et al. Blinatumomab versus Chemotherapy for Advanced Acute Lymphoblastic Leukemia. *N Engl J Med*. Mar 2 2017;376(9):836-847. doi:10.1056/NEJMoa1609783

62. Coiffier B, Osmanov EA, Hong X, et al. Bortezomib plus rituximab versus rituximab alone in patients with relapsed, rituximab-naive or rituximab-sensitive, follicular lymphoma: a randomised phase 3 trial. *Lancet Oncol*. Aug 2011;12(8):773-84. doi:10.1016/s1470-2045(11)70150-4

63. Moskowitz CH, Nademanee A, Masszi T, et al. Brentuximab vedotin as consolidation therapy after autologous stem-cell transplantation in patients with Hodgkin's lymphoma at risk of relapse or progression (AETHERA): a randomised, double-blind, placebo-controlled, phase 3 trial. *Lancet*. May 9 2015;385(9980):1853-62. doi:10.1016/s0140-6736(15)60165-9

64. Prince HM, Kim YH, Horwitz SM, et al. Brentuximab vedotin or physician's choice in CD30-positive cutaneous T-cell lymphoma (ALCANZA): an international, open-label, randomised, phase 3, multicentre trial. *Lancet*. Aug 5 2017;390(10094):555-566. doi:10.1016/s0140-6736(17)31266-7

65. Horwitz S, O'Connor OA, Pro B, et al. Brentuximab vedotin with chemotherapy for CD30-positive peripheral T-cell lymphoma (ECHELON-2): a global, double-blind, randomised, phase 3 trial. *Lancet*. Jan 19 2019;393(10168):229-240. doi:10.1016/s0140-6736(18)32984-2

66. Connors JM, Jurczak W, Straus DJ, et al. Brentuximab Vedotin with Chemotherapy for Stage III or IV Hodgkin's Lymphoma. *N Engl J Med*. Jan 25 2018;378(4):331-344. doi:10.1056/NEJMoa1708984

67. Di Leo A, Johnston S, Lee KS, et al. Buparlisib plus fulvestrant in postmenopausal women with hormone-receptor-positive, HER2-negative, advanced breast cancer progressing on or after mTOR inhibition (BELLE-3): a randomised, double-blind, placebo-controlled, phase 3 trial. *Lancet Oncol*. Jan 2018;19(1):87-100. doi:10.1016/s1470-2045(17)30688-5

© 2024 Sherry AD et al. *JAMA Network Open.*

68. Abou-Alfa GK, Meyer T, Cheng AL, et al. Cabozantinib in Patients with Advanced and Progressing Hepatocellular Carcinoma. *N Engl J Med*. Jul 5 2018;379(1):54-63. doi:10.1056/NEJMoa1717002

69. Elisei R, Schlumberger MJ, Müller SP, et al. Cabozantinib in progressive medullary thyroid cancer. *J Clin Oncol*. Oct 10 2013;31(29):3639-46. doi:10.1200/jco.2012.48.4659

70. Lordick F, Kang YK, Chung HC, et al. Capecitabine and cisplatin with or without cetuximab for patients with previously untreated advanced gastric cancer (EXPAND): a randomised, open-label phase 3 trial. *Lancet Oncol*. May 2013;14(6):490-9. doi:10.1016/s1470-2045(13)70102-5

71. Twelves C, Wong A, Nowacki MP, et al. Capecitabine as adjuvant treatment for stage III colon cancer. *N Engl J Med*. Jun 30 2005;352(26):2696-704. doi:10.1056/NEJMoa043116

72. Rothenberg ML, Cox JV, Butts C, et al. Capecitabine plus oxaliplatin (XELOX) versus 5-fluorouracil/folinic acid plus oxaliplatin (FOLFOX-4) as second-line therapy in metastatic colorectal cancer: a randomized phase III noninferiority study. *Ann Oncol*. Oct 2008;19(10):1720-6. doi:10.1093/annonc/mdn370

73. Haller DG, Tabernero J, Maroun J, et al. Capecitabine plus oxaliplatin compared with fluorouracil and folinic acid as adjuvant therapy for stage III colon cancer. *J Clin Oncol*. Apr 10 2011;29(11):1465-71. doi:10.1200/jco.2010.33.6297

74. Dimopoulos MA, Moreau P, Palumbo A, et al. Carfilzomib and dexamethasone versus bortezomib and dexamethasone for patients with relapsed or refractory multiple myeloma (ENDEAVOR): a randomised, phase 3, open-label, multicentre study. *Lancet Oncol*. Jan 2016;17(1):27-38. doi:10.1016/s1470-2045(15)00464-7

75. Facon T, Lee JH, Moreau P, et al. Carfilzomib or bortezomib with melphalan-prednisone for transplant-ineligible patients with newly diagnosed multiple myeloma. *Blood*. May 2 2019;133(18):1953-1963. doi:10.1182/blood-2018-09-874396

76. Stewart AK, Rajkumar SV, Dimopoulos MA, et al. Carfilzomib, lenalidomide, and dexamethasone for relapsed multiple myeloma. *N Engl J Med*. Jan 8 2015;372(2):142-52. doi:10.1056/NEJMoa1411321

© 2024 Sherry AD et al. *JAMA Network Open.*

77. Hoff PM, Hochhaus A, Pestalozzi BC, et al. Cediranib plus FOLFOX/CAPOX versus placebo plus FOLFOX/CAPOX in patients with previously untreated metastatic colorectal cancer: a randomized, double-blind, phase III study (HORIZON II). *J Clin Oncol*. Oct 10 2012;30(29):3596-603. doi:10.1200/jco.2012.42.6031

78. Schmoll HJ, Cunningham D, Sobrero A, et al. Cediranib with mFOLFOX6 versus bevacizumab with mFOLFOX6 as first-line treatment for patients with advanced colorectal cancer: a double-blind, randomized phase III study (HORIZON III). *J Clin Oncol*. Oct 10 2012;30(29):3588-95. doi:10.1200/jco.2012.42.5355

79. Shaw AT, Kim TM, Crinò L, et al. Ceritinib versus chemotherapy in patients with ALK-rearranged non-small-cell lung cancer previously given chemotherapy and crizotinib (ASCEND-5): a randomised, controlled, open-label, phase 3 trial. *Lancet Oncol*. Jul 2017;18(7):874-886. doi:10.1016/s1470-2045(17)30339-x

80. Van Cutsem E, Köhne CH, Hitre E, et al. Cetuximab and chemotherapy as initial treatment for metastatic colorectal cancer. *N Engl J Med*. Apr 2 2009;360(14):1408-17. doi:10.1056/NEJMoa0805019

81. Lynch TJ, Patel T, Dreisbach L, et al. Cetuximab and first-line taxane/carboplatin chemotherapy in advanced non-small-cell lung cancer: results of the randomized multicenter phase III trial BMS099. *J Clin Oncol*. Feb 20 2010;28(6):911-7. doi:10.1200/jco.2009.21.9618

82. Herbst RS, Redman MW, Kim ES, et al. Cetuximab plus carboplatin and paclitaxel with or without bevacizumab versus carboplatin and paclitaxel with or without bevacizumab in advanced NSCLC (SWOG S0819): a randomised, phase 3 study. *Lancet Oncol*. Jan 2018;19(1):101-114. doi:10.1016/s1470-2045(17)30694-0

83. Pirker R, Pereira JR, Szczesna A, et al. Cetuximab plus chemotherapy in patients with advanced non-small-cell lung cancer (FLEX): an open-label randomised phase III trial. *Lancet*. May 2 2009;373(9674):1525-31. doi:10.1016/s0140-6736(09)60569-9

84. Sweeney CJ, Chen YH, Carducci M, et al. Chemohormonal Therapy in Metastatic Hormone-Sensitive Prostate Cancer. *N Engl J Med*. Aug 20 2015;373(8):737-46. doi:10.1056/NEJMoa1503747

85. Aebi S, Gelber S, Anderson SJ, et al. Chemotherapy for isolated locoregional recurrence of breast cancer (CALOR): a randomised trial. *Lancet Oncol*. Feb 2014;15(2):156-63. doi:10.1016/s1470-2045(13)70589-8

© 2024 Sherry AD et al. *JAMA Network Open.*

86. Hillmen P, Robak T, Janssens A, et al. Chlorambucil plus ofatumumab versus chlorambucil alone in previously untreated patients with chronic lymphocytic leukaemia (COMPLEMENT 1): a randomised, multicentre, open-label phase 3 trial. *Lancet*. May 9 2015;385(9980):1873-83. doi:10.1016/s0140-6736(15)60027-7

87. Stupp R, Hegi ME, Gorlia T, et al. Cilengitide combined with standard treatment for patients with newly diagnosed glioblastoma with methylated MGMT promoter (CENTRIC EORTC 26071-22072 study): a multicentre, randomised, open-label, phase 3 trial. *Lancet Oncol*. Sep 2014;15(10):1100-8. doi:10.1016/s1470-2045(14)70379-1

88. Vermorken JB, Stöhlmacher-Williams J, Davidenko I, et al. Cisplatin and fluorouracil with or without panitumumab in patients with recurrent or metastatic squamous-cell carcinoma of the head and neck (SPECTRUM): an open-label phase 3 randomised trial. *Lancet Oncol*. Jul 2013;14(8):697-710. doi:10.1016/s1470-2045(13)70181-5

89. Mehta RS, Barlow WE, Albain KS, et al. Combination anastrozole and fulvestrant in metastatic breast cancer. *N Engl J Med*. Aug 2 2012;367(5):435-44. doi:10.1056/NEJMoa1201622

90. Long GV, Stroyakovskiy D, Gogas H, et al. Combined BRAF and MEK inhibition versus BRAF inhibition alone in melanoma. *N Engl J Med*. Nov 13 2014;371(20):1877-88. doi:10.1056/NEJMoa1406037

91. Larkin J, Ascierto PA, Dréno B, et al. Combined vemurafenib and cobimetinib in BRAF-mutated melanoma. *N Engl J Med*. Nov 13 2014;371(20):1867-76. doi:10.1056/NEJMoa1408868

92. Rini BI, Escudier B, Tomczak P, et al. Comparative effectiveness of axitinib versus sorafenib in advanced renal cell carcinoma (AXIS): a randomised phase 3 trial. *Lancet*. Dec 3 2011;378(9807):1931-9. doi:10.1016/s0140-6736(11)61613-9

93. Smith I, Yardley D, Burris H, et al. Comparative Efficacy and Safety of Adjuvant Letrozole Versus Anastrozole in Postmenopausal Patients With Hormone Receptor-Positive, Node-Positive Early Breast Cancer: Final Results of the Randomized Phase III Femara Versus Anastrozole Clinical Evaluation (FACE) Trial. *J Clin Oncol*. Apr 1 2017;35(10):1041-1048. doi:10.1200/jco.2016.69.2871

94. Bennouna J, Sastre J, Arnold D, et al. Continuation of bevacizumab after first progression in metastatic colorectal cancer (ML18147): a randomised phase 3 trial. *Lancet Oncol*. Jan 2013;14(1):29-37. doi:10.1016/s1470-2045(12)70477-1

© 2024 Sherry AD et al. *JAMA Network Open.*

95. Lancet JE, Uy GL, Cortes JE, et al. CPX-351 (cytarabine and daunorubicin) Liposome for Injection Versus Conventional Cytarabine Plus Daunorubicin in Older Patients With Newly Diagnosed Secondary Acute Myeloid Leukemia. *J Clin Oncol*. Sep 10 2018;36(26):2684-2692. doi:10.1200/jco.2017.77.6112

96. Ellis PM, Shepherd FA, Millward M, et al. Dacomitinib compared with placebo in pretreated patients with advanced or metastatic non-small-cell lung cancer (NCIC CTG BR.26): a double-blind, randomised, phase 3 trial. *Lancet Oncol*. Nov 2014;15(12):1379-88. doi:10.1016/s1470-2045(14)70472-3

97. Ramalingam SS, Jänne PA, Mok T, et al. Dacomitinib versus erlotinib in patients with advanced-stage, previously treated non-small-cell lung cancer (ARCHER 1009): a randomised, double-blind, phase 3 trial. *Lancet Oncol*. Nov 2014;15(12):1369-78. doi:10.1016/s1470-2045(14)70452-8

98. Wu YL, Cheng Y, Zhou X, et al. Dacomitinib versus gefitinib as first-line treatment for patients with EGFR-mutation-positive non-small-cell lung cancer (ARCHER 1050): a randomised, open-label, phase 3 trial. *Lancet Oncol*. Nov 2017;18(11):1454-1466. doi:10.1016/s1470-2045(17)30608-3

99. Mateos MV, Dimopoulos MA, Cavo M, et al. Daratumumab plus Bortezomib, Melphalan, and Prednisone for Untreated Myeloma. *N Engl J Med*. Feb 8 2018;378(6):518-528. doi:10.1056/NEJMoa1714678

100. Facon T, Kumar S, Plesner T, et al. Daratumumab plus Lenalidomide and Dexamethasone for Untreated Myeloma. *N Engl J Med*. May 30 2019;380(22):2104-2115. doi:10.1056/NEJMoa1817249

101. Palumbo A, Chanan-Khan A, Weisel K, et al. Daratumumab, Bortezomib, and Dexamethasone for Multiple Myeloma. *N Engl J Med*. Aug 25 2016;375(8):754-66. doi:10.1056/NEJMoa1606038

102. Dimopoulos MA, Oriol A, Nahi H, et al. Daratumumab, Lenalidomide, and Dexamethasone for Multiple Myeloma. *N Engl J Med*. Oct 6 2016;375(14):1319-1331. doi:10.1056/NEJMoa1607751

103. Fizazi K, Shore N, Tammela TL, et al. Darolutamide in Nonmetastatic, Castration-Resistant Prostate Cancer. *N Engl J Med*. Mar 28 2019;380(13):1235-1246. doi:10.1056/NEJMoa1815671

© 2024 Sherry AD et al. *JAMA Network Open.*

104. Swain SM, Tang G, Geyer CE, Jr., et al. Definitive results of a phase III adjuvant trial comparing three chemotherapy regimens in women with operable, node-positive breast cancer: the NSABP B-38 trial. *J Clin Oncol*. Sep 10 2013;31(26):3197-204. doi:10.1200/jco.2012.48.1275

105. Araujo JC, Trudel GC, Saad F, et al. Docetaxel and dasatinib or placebo in men with metastatic castration-resistant prostate cancer (READY): a randomised, double-blind phase 3 trial. *Lancet Oncol*. Dec 2013;14(13):1307-16. doi:10.1016/s1470-2045(13)70479-0

106. Kim ES, Neubauer M, Cohn A, et al. Docetaxel or pemetrexed with or without cetuximab in recurrent or progressive non-small-cell lung cancer after platinum-based therapy: a phase 3, open-label, randomised trial. *Lancet Oncol*. Dec 2013;14(13):1326-36. doi:10.1016/s1470-2045(13)70473-x

107. Reck M, Kaiser R, Mellemgaard A, et al. Docetaxel plus nintedanib versus docetaxel plus placebo in patients with previously treated non-small-cell lung cancer (LUME-Lung 1): a phase 3, double-blind, randomised controlled trial. *Lancet Oncol*. Feb 2014;15(2):143-55. doi:10.1016/s1470-2045(13)70586-2

108. Kurzeder C, Bover I, Marmé F, et al. Double-Blind, Placebo-Controlled, Randomized Phase III Trial Evaluating Pertuzumab Combined With Chemotherapy for Low Tumor Human Epidermal Growth Factor Receptor 3 mRNA-Expressing Platinum-Resistant Ovarian Cancer (PENELOPE). *J Clin Oncol*. Jul 20 2016;34(21):2516-25. doi:10.1200/jco.2015.66.0787

109. Pisansky TM, Hunt D, Gomella LG, et al. Duration of androgen suppression before radiotherapy for localized prostate cancer: radiation therapy oncology group randomized clinical trial 9910. *J Clin Oncol*. Feb 1 2015;33(4):332-9. doi:10.1200/jco.2014.58.0662

110. Antonia SJ, Villegas A, Daniel D, et al. Durvalumab after Chemoradiotherapy in Stage III Non-Small-Cell Lung Cancer. *N Engl J Med*. Nov 16 2017;377(20):1919-1929. doi:10.1056/NEJMoa1709937

111. Zhu AX, Kudo M, Assenat E, et al. Effect of everolimus on survival in advanced hepatocellular carcinoma after failure of sorafenib: the EVOLVE-1 randomized clinical trial. *Jama*. Jul 2 2014;312(1):57-67. doi:10.1001/jama.2014.7189

112. Siu LL, Waldron JN, Chen BE, et al. Effect of Standard Radiotherapy With Cisplatin vs Accelerated Radiotherapy With Panitumumab in Locoregionally Advanced

Squamous Cell Head and Neck Carcinoma: A Randomized Clinical Trial. *JAMA Oncol*. Feb 1 2017;3(2):220-226. doi:10.1001/jamaoncol.2016.4510

113. Ciuleanu T, Stelmakh L, Cicenas S, et al. Efficacy and safety of erlotinib versus chemotherapy in second-line treatment of patients with advanced, non-small-cell lung cancer with poor prognosis (TITAN): a randomised multicentre, open-label, phase 3 study. *Lancet Oncol*. Mar 2012;13(3):300-8. doi:10.1016/s1470-2045(11)70385-0

114. Jones JA, Robak T, Brown JR, et al. Efficacy and safety of idelalisib in combination with ofatumumab for previously treated chronic lymphocytic leukaemia: an open-label, randomised phase 3 trial. *Lancet Haematol*. Mar 2017;4(3):e114-e126. doi:10.1016/s2352-3026(17)30019-4

115. Demetri GD, Reichardt P, Kang YK, et al. Efficacy and safety of regorafenib for advanced gastrointestinal stromal tumours after failure of imatinib and sunitinib (GRID): an international, multicentre, randomised, placebo-controlled, phase 3 trial. *Lancet*. Jan 26 2013;381(9863):295-302. doi:10.1016/s0140-6736(12)61857-1

116. Cheng AL, Kang YK, Chen Z, et al. Efficacy and safety of sorafenib in patients in the Asia-Pacific region with advanced hepatocellular carcinoma: a phase III randomised, double-blind, placebo-controlled trial. *Lancet Oncol*. Jan 2009;10(1):25-34. doi:10.1016/s1470-2045(08)70285-7

117. Demetri GD, van Oosterom AT, Garrett CR, et al. Efficacy and safety of sunitinib in patients with advanced gastrointestinal stromal tumour after failure of imatinib: a randomised controlled trial. *Lancet*. Oct 14 2006;368(9544):1329-38. doi:10.1016/s0140-6736(06)69446-4

118. Demetri GD, von Mehren M, Jones RL, et al. Efficacy and Safety of Trabectedin or Dacarbazine for Metastatic Liposarcoma or Leiomyosarcoma After Failure of Conventional Chemotherapy: Results of a Phase III Randomized Multicenter Clinical Trial. *J Clin Oncol*. Mar 10 2016;34(8):786-93. doi:10.1200/jco.2015.62.4734

119. Qin S, Li J, Wang L, et al. Efficacy and Tolerability of First-Line Cetuximab Plus Leucovorin, Fluorouracil, and Oxaliplatin (FOLFOX-4) Versus FOLFOX-4 in Patients With RAS Wild-Type Metastatic Colorectal Cancer: The Open-Label, Randomized, Phase III TAILOR Trial. *J Clin Oncol*. Oct 20 2018;36(30):3031-3039. doi:10.1200/jco.2018.78.3183

120. Fenaux P, Mufti GJ, Hellstrom-Lindberg E, et al. Efficacy of azacitidine compared with that of conventional care regimens in the treatment of higher-risk myelodysplastic

syndromes: a randomised, open-label, phase III study. *Lancet Oncol*. Mar 2009;10(3):223-32. doi:10.1016/s1470-2045(09)70003-8

121. Herbst RS, Ansari R, Bustin F, et al. Efficacy of bevacizumab plus erlotinib versus erlotinib alone in advanced non-small-cell lung cancer after failure of standard first-line chemotherapy (BeTa): a double-blind, placebo-controlled, phase 3 trial. *Lancet*. May 28 2011;377(9780):1846-54. doi:10.1016/s0140-6736(11)60545-x

122. Motzer RJ, Escudier B, Oudard S, et al. Efficacy of everolimus in advanced renal cell carcinoma: a double-blind, randomised, placebo-controlled phase III trial. *Lancet*. Aug 9 2008;372(9637):449-56. doi:10.1016/s0140-6736(08)61039-9

123. Vansteenkiste JF, Cho BC, Vanakesa T, et al. Efficacy of the MAGE-A3 cancer immunotherapeutic as adjuvant therapy in patients with resected MAGE-A3-positive non-small-cell lung cancer (MAGRIT): a randomised, double-blind, placebo-controlled, phase 3 trial. *Lancet Oncol*. Jun 2016;17(6):822-835. doi:10.1016/s1470-2045(16)00099-1

124. Lonial S, Dimopoulos M, Palumbo A, et al. Elotuzumab Therapy for Relapsed or Refractory Multiple Myeloma. *N Engl J Med*. Aug 13 2015;373(7):621-31. doi:10.1056/NEJMoa1505654

125. Kang YK, Yook JH, Chang HM, et al. Enhanced efficacy of postoperative adjuvant chemotherapy in advanced gastric cancer: results from a phase 3 randomized trial (AMC0101). *Cancer Chemother Pharmacol*. Jan 2014;73(1):139-49. doi:10.1007/s00280-013-2332-5

126. Long GV, Dummer R, Hamid O, et al. Epacadostat plus pembrolizumab versus placebo plus pembrolizumab in patients with unresectable or metastatic melanoma (ECHO-301/KEYNOTE-252): a phase 3, randomised, double-blind study. *Lancet Oncol*. Aug 2019;20(8):1083-1097. doi:10.1016/s1470-2045(19)30274-8

127. Martín M, Ruiz Simón A, Ruiz Borrego M, et al. Epirubicin Plus Cyclophosphamide Followed by Docetaxel Versus Epirubicin Plus Docetaxel Followed by Capecitabine As Adjuvant Therapy for Node-Positive Early Breast Cancer: Results From the GEICAM/2003-10 Study. *J Clin Oncol*. Nov 10 2015;33(32):3788-95. doi:10.1200/jco.2015.61.9510

128. Cortes J, O'Shaughnessy J, Loesch D, et al. Eribulin monotherapy versus treatment of physician's choice in patients with metastatic breast cancer (EMBRACE): a

© 2024 Sherry AD et al. *JAMA Network Open.*

phase 3 open-label randomised study. *Lancet*. Mar 12 2011;377(9769):914-23. doi:10.1016/s0140-6736(11)60070-6

129. Schöffski P, Chawla S, Maki RG, et al. Eribulin versus dacarbazine in previously treated patients with advanced liposarcoma or leiomyosarcoma: a randomised, open-label, multicentre, phase 3 trial. *Lancet*. Apr 16 2016;387(10028):1629-37. doi:10.1016/s0140-6736(15)01283-0

130. Cappuzzo F, Ciuleanu T, Stelmakh L, et al. Erlotinib as maintenance treatment in advanced non-small-cell lung cancer: a multicentre, randomised, placebo-controlled phase 3 study. *Lancet Oncol*. Jun 2010;11(6):521-9. doi:10.1016/s1470-2045(10)70112-1

131. Martín M, Loibl S, Hyslop T, et al. Evaluating the addition of bevacizumab to endocrine therapy as first-line treatment for hormone receptor-positive metastatic breast cancer: a pooled analysis from the LEA (GEICAM/2006-11_GBG51) and CALGB 40503 (Alliance) trials. *Eur J Cancer*. Aug 2019;117:91-98. doi:10.1016/j.ejca.2019.06.002

132. Bookman MA, Brady MF, McGuire WP, et al. Evaluation of new platinum-based treatment regimens in advanced-stage ovarian cancer: a Phase III Trial of the Gynecologic Cancer Intergroup. *J Clin Oncol*. Mar 20 2009;27(9):1419-25. doi:10.1200/jco.2008.19.1684

133. Yao JC, Shah MH, Ito T, et al. Everolimus for advanced pancreatic neuroendocrine tumors. *N Engl J Med*. Feb 10 2011;364(6):514-23. doi:10.1056/NEJMoa1009290

134. Bissler JJ, Kingswood JC, Radzikowska E, et al. Everolimus for angiomyolipoma associated with tuberous sclerosis complex or sporadic lymphangioleiomyomatosis (EXIST-2): a multicentre, randomised, double-blind, placebo-controlled trial. *Lancet*. Mar 9 2013;381(9869):817-24. doi:10.1016/s0140-6736(12)61767-x

135. Ohtsu A, Ajani JA, Bai YX, et al. Everolimus for previously treated advanced gastric cancer: results of the randomized, double-blind, phase III GRANITE-1 study. *J Clin Oncol*. Nov 1 2013;31(31):3935-43. doi:10.1200/jco.2012.48.3552

136. Yao JC, Fazio N, Singh S, et al. Everolimus for the treatment of advanced, non-functional neuroendocrine tumours of the lung or gastrointestinal tract (RADIANT-4): a randomised, placebo-controlled, phase 3 study. *Lancet*. Mar 5 2016;387(10022):968-977. doi:10.1016/s0140-6736(15)00817-x

© 2024 Sherry AD et al. *JAMA Network Open.*

137. André F, O'Regan R, Ozguroglu M, et al. Everolimus for women with trastuzumab-resistant, HER2-positive, advanced breast cancer (BOLERO-3): a randomised, double-blind, placebo-controlled phase 3 trial. *Lancet Oncol*. May 2014;15(6):580-91. doi:10.1016/s1470-2045(14)70138-x

138. Baselga J, Campone M, Piccart M, et al. Everolimus in postmenopausal hormone-receptor-positive advanced breast cancer. *N Engl J Med*. Feb 9 2012;366(6):520-9. doi:10.1056/NEJMoa1109653

139. Pavel ME, Hainsworth JD, Baudin E, et al. Everolimus plus octreotide long-acting repeatable for the treatment of advanced neuroendocrine tumours associated with carcinoid syndrome (RADIANT-2): a randomised, placebo-controlled, phase 3 study. *Lancet*. Dec 10 2011;378(9808):2005-2012. doi:10.1016/s0140-6736(11)61742-x

140. Colleoni M, Luo W, Karlsson P, et al. Extended adjuvant intermittent letrozole versus continuous letrozole in postmenopausal women with breast cancer (SOLE): a multicentre, open-label, randomised, phase 3 trial. *Lancet Oncol*. Jan 2018;19(1):127-138. doi:10.1016/s1470-2045(17)30715-5

141. Bergh J, Jönsson PE, Lidbrink EK, et al. FACT: an open-label randomized phase III study of fulvestrant and anastrozole in combination compared with anastrozole alone as first-line therapy for patients with receptor-positive postmenopausal breast cancer. *J Clin Oncol*. Jun 1 2012;30(16):1919-25. doi:10.1200/jco.2011.38.1095

142. Horn L, Mansfield AS, Szczęsna A, et al. First-Line Atezolizumab plus Chemotherapy in Extensive-Stage Small-Cell Lung Cancer. *N Engl J Med*. Dec 6 2018;379(23):2220-2229. doi:10.1056/NEJMoa1809064

143. Soria JC, Tan DSW, Chiari R, et al. First-line ceritinib versus platinum-based chemotherapy in advanced ALK-rearranged non-small-cell lung cancer (ASCEND-4): a randomised, open-label, phase 3 study. *Lancet*. Mar 4 2017;389(10072):917-929. doi:10.1016/s0140-6736(17)30123-x

144. Solomon BJ, Mok T, Kim DW, et al. First-line crizotinib versus chemotherapy in ALK-positive lung cancer. *N Engl J Med*. Dec 4 2014;371(23):2167-77. doi:10.1056/NEJMoa1408440

145. Wu YL, Zhou C, Liam CK, et al. First-line erlotinib versus gemcitabine/cisplatin in patients with advanced EGFR mutation-positive non-small-cell lung cancer: analyses from the phase III, randomized, open-label, ENSURE study. *Ann Oncol*. Sep 2015;26(9):1883-1889. doi:10.1093/annonc/mdv270

146. Carbone DP, Reck M, Paz-Ares L, et al. First-Line Nivolumab in Stage IV or Recurrent Non-Small-Cell Lung Cancer. *N Engl J Med*. Jun 22 2017;376(25):2415-2426. doi:10.1056/NEJMoa1613493

147. Yang JC, Kang JH, Mok T, et al. First-line pemetrexed plus cisplatin followed by gefitinib maintenance therapy versus gefitinib monotherapy in East Asian patients with locally advanced or metastatic non-squamous non-small cell lung cancer: a randomised, phase 3 trial. *Eur J Cancer*. Sep 2014;50(13):2219-30. doi:10.1016/j.ejca.2014.05.011

148. Wasan HS, Gibbs P, Sharma NK, et al. First-line selective internal radiotherapy plus chemotherapy versus chemotherapy alone in patients with liver metastases from colorectal cancer (FOXFIRE, SIRFLOX, and FOXFIRE-Global): a combined analysis of three multicentre, randomised, phase 3 trials. *Lancet Oncol*. Sep 2017;18(9):1159-1171. doi:10.1016/s1470-2045(17)30457-6

149. Bergh J, Bondarenko IM, Lichinitser MR, et al. First-line treatment of advanced breast cancer with sunitinib in combination with docetaxel versus docetaxel alone: results of a prospective, randomized phase III study. *J Clin Oncol*. Mar 20 2012;30(9):921-9. doi:10.1200/jco.2011.35.7376

150. Spina M, Nagy Z, Ribera JM, et al. FLORENCE: a randomized, double-blind, phase III pivotal study of febuxostat versus allopurinol for the prevention of tumor lysis syndrome (TLS) in patients with hematologic malignancies at intermediate to high TLS risk. *Ann Oncol*. Oct 2015;26(10):2155-61. doi:10.1093/annonc/mdv317

151. Kornblit B, Maloney DG, Storb R, et al. Fludarabine and 2-Gy TBI is superior to 2 Gy TBI as conditioning for HLA-matched related hematopoietic cell transplantation: a phase III randomized trial. *Biol Blood Marrow Transplant*. Sep 2013;19(9):1340-7. doi:10.1016/j.bbmt.2013.06.002

152. Elter T, Gercheva-Kyuchukova L, Pylylpenko H, et al. Fludarabine plus alemtuzumab versus fludarabine alone in patients with previously treated chronic lymphocytic leukaemia: a randomised phase 3 trial. *Lancet Oncol*. Dec 2011;12(13):1204-13. doi:10.1016/s1470-2045(11)70242-x

153. Robertson JFR, Bondarenko IM, Trishkina E, et al. Fulvestrant 500 mg versus anastrozole 1 mg for hormone receptor-positive advanced breast cancer (FALCON): an international, randomised, double-blind, phase 3 trial. *Lancet*. Dec 17 2016;388(10063):2997-3005. doi:10.1016/s0140-6736(16)32389-3

© 2024 Sherry AD et al. *JAMA Network Open.*

154. Zhang Q, Shao Z, Shen K, et al. Fulvestrant 500 mg vs 250 mg in postmenopausal women with estrogen receptor-positive advanced breast cancer: a randomized, double-blind registrational trial in China. *Oncotarget*. Aug 30 2016;7(35):57301-57309. doi:10.18632/oncotarget.10254

155. Soria JC, Wu YL, Nakagawa K, et al. Gefitinib plus chemotherapy versus placebo plus chemotherapy in EGFR-mutation-positive non-small-cell lung cancer after progression on first-line gefitinib (IMPRESS): a phase 3 randomised trial. *Lancet Oncol*. Aug 2015;16(8):990-8. doi:10.1016/s1470-2045(15)00121-7

156. Perl AE, Martinelli G, Cortes JE, et al. Gilteritinib or Chemotherapy for Relapsed or Refractory FLT3-Mutated AML. *N Engl J Med*. Oct 31 2019;381(18):1728-1740. doi:10.1056/NEJMoa1902688

157. Shah MA, Xu RH, Bang YJ, et al. HELOISE: Phase IIIb Randomized Multicenter Study Comparing Standard-of-Care and Higher-Dose Trastuzumab Regimens Combined With Chemotherapy as First-Line Therapy in Patients With Human Epidermal Growth Factor Receptor 2-Positive Metastatic Gastric or Gastroesophageal Junction Adenocarcinoma. *J Clin Oncol*. Aug 1 2017;35(22):2558-2567. doi:10.1200/jco.2016.71.6852

158. Zapatero A, Guerrero A, Maldonado X, et al. High-dose radiotherapy with short-term or long-term androgen deprivation in localised prostate cancer (DART01/05 GICOR): a randomised, controlled, phase 3 trial. *Lancet Oncol*. Mar 2015;16(3):320-7. doi:10.1016/s1470-2045(15)70045-8

159. Burger JA, Tedeschi A, Barr PM, et al. Ibrutinib as Initial Therapy for Patients with Chronic Lymphocytic Leukemia. *N Engl J Med*. Dec 17 2015;373(25):2425-37. doi:10.1056/NEJMoa1509388

160. Moreno C, Greil R, Demirkan F, et al. Ibrutinib plus obinutuzumab versus chlorambucil plus obinutuzumab in first-line treatment of chronic lymphocytic leukaemia (iLLUMINATE): a multicentre, randomised, open-label, phase 3 trial. *Lancet Oncol*. Jan 2019;20(1):43-56. doi:10.1016/s1470-2045(18)30788-5

161. Woyach JA, Ruppert AS, Heerema NA, et al. Ibrutinib Regimens versus Chemoimmunotherapy in Older Patients with Untreated CLL. *N Engl J Med*. Dec 27 2018;379(26):2517-2528. doi:10.1056/NEJMoa1812836

© 2024 Sherry AD et al. *JAMA Network Open.*

162. Byrd JC, Brown JR, O'Brien S, et al. Ibrutinib versus ofatumumab in previously treated chronic lymphoid leukemia. *N Engl J Med*. Jul 17 2014;371(3):213-23. doi:10.1056/NEJMoa1400376

163. Huang X, Qiu L, Jin J, et al. Ibrutinib versus rituximab in relapsed or refractory chronic lymphocytic leukemia or small lymphocytic lymphoma: a randomized, open-label phase 3 study. *Cancer Med*. Apr 2018;7(4):1043-1055. doi:10.1002/cam4.1337

164. Dreyling M, Jurczak W, Jerkeman M, et al. Ibrutinib versus temsirolimus in patients with relapsed or refractory mantle-cell lymphoma: an international, randomised, open-label, phase 3 study. *Lancet*. Feb 20 2016;387(10020):770-8. doi:10.1016/s0140-6736(15)00667-4

165. Shi Y, Zhang L, Liu X, et al. Icotinib versus gefitinib in previously treated advanced non-small-cell lung cancer (ICOGEN): a randomised, double-blind phase 3 non-inferiority trial. *Lancet Oncol*. Sep 2013;14(10):953-61. doi:10.1016/s1470-2045(13)70355-3

166. Furman RR, Sharman JP, Coutre SE, et al. Idelalisib and rituximab in relapsed chronic lymphocytic leukemia. *N Engl J Med*. Mar 13 2014;370(11):997-1007. doi:10.1056/NEJMoa1315226

167. Zelenetz AD, Barrientos JC, Brown JR, et al. Idelalisib or placebo in combination with bendamustine and rituximab in patients with relapsed or refractory chronic lymphocytic leukaemia: interim results from a phase 3, randomised, double-blind, placebo-controlled trial. *Lancet Oncol*. Mar 2017;18(3):297-311. doi:10.1016/s1470-2045(16)30671-4

168. Fernando HC, Landreneau RJ, Mandrekar SJ, et al. Impact of brachytherapy on local recurrence rates after sublobar resection: results from ACOSOG Z4032 (Alliance), a phase III randomized trial for high-risk operable non-small-cell lung cancer. *J Clin Oncol*. Aug 10 2014;32(23):2456-62. doi:10.1200/jco.2013.53.4115

169. Robert C, Karaszewska B, Schachter J, et al. Improved overall survival in melanoma with combined dabrafenib and trametinib. *N Engl J Med*. Jan 1 2015;372(1):30-9. doi:10.1056/NEJMoa1412690

170. Tewari KS, Sill MW, Long HJ, 3rd, et al. Improved survival with bevacizumab in advanced cervical cancer. *N Engl J Med*. Feb 20 2014;370(8):734-43. doi:10.1056/NEJMoa1309748

171. Hodi FS, O'Day SJ, McDermott DF, et al. Improved survival with ipilimumab in patients with metastatic melanoma. *N Engl J Med*. Aug 19 2010;363(8):711-23. doi:10.1056/NEJMoa1003466

172. Flaherty KT, Robert C, Hersey P, et al. Improved survival with MEK inhibition in BRAF-mutated melanoma. *N Engl J Med*. Jul 12 2012;367(2):107-14. doi:10.1056/NEJMoa1203421

173. Chapman PB, Hauschild A, Robert C, et al. Improved survival with vemurafenib in melanoma with BRAF V600E mutation. *N Engl J Med*. Jun 30 2011;364(26):2507-16. doi:10.1056/NEJMoa1103782

174. Burger RA, Brady MF, Bookman MA, et al. Incorporation of bevacizumab in the primary treatment of ovarian cancer. *N Engl J Med*. Dec 29 2011;365(26):2473-83. doi:10.1056/NEJMoa1104390

175. Von Hoff DD, Ervin T, Arena FP, et al. Increased survival in pancreatic cancer with nab-paclitaxel plus gemcitabine. *N Engl J Med*. Oct 31 2013;369(18):1691-703. doi:10.1056/NEJMoa1304369

176. Scher HI, Fizazi K, Saad F, et al. Increased survival with enzalutamide in prostate cancer after chemotherapy. *N Engl J Med*. Sep 27 2012;367(13):1187-97. doi:10.1056/NEJMoa1207506

177. Haddad R, O'Neill A, Rabinowits G, et al. Induction chemotherapy followed by concurrent chemoradiotherapy (sequential chemoradiotherapy) versus concurrent chemoradiotherapy alone in locally advanced head and neck cancer (PARADIGM): a randomised phase 3 trial. *Lancet Oncol*. Mar 2013;14(3):257-64. doi:10.1016/s1470-2045(13)70011-1

178. Wu YL, Lee JS, Thongprasert S, et al. Intercalated combination of chemotherapy and erlotinib for patients with advanced stage non-small-cell lung cancer (FASTACT-2): a randomised, double-blind trial. *Lancet Oncol*. Jul 2013;14(8):777-86. doi:10.1016/s1470-2045(13)70254-7

179. Hussain M, Tangen CM, Berry DL, et al. Intermittent versus continuous androgen deprivation in prostate cancer. *N Engl J Med*. Apr 4 2013;368(14):1314-25. doi:10.1056/NEJMoa1212299

© 2024 Sherry AD et al. *JAMA Network Open.*

180. Dombret H, Seymour JF, Butrym A, et al. International phase 3 study of azacitidine vs conventional care regimens in older patients with newly diagnosed AML with >30% blasts. *Blood*. Jul 16 2015;126(3):291-9. doi:10.1182/blood-2015-01-621664

181. Ascierto PA, Del Vecchio M, Robert C, et al. Ipilimumab 10 mg/kg versus ipilimumab 3 mg/kg in patients with unresectable or metastatic melanoma: a randomised, double-blind, multicentre, phase 3 trial. *Lancet Oncol*. May 2017;18(5):611-622. doi:10.1016/s1470-2045(17)30231-0

182. Robert C, Thomas L, Bondarenko I, et al. Ipilimumab plus dacarbazine for previously untreated metastatic melanoma. *N Engl J Med*. Jun 30 2011;364(26):2517-26. doi:10.1056/NEJMoa1104621

183. Kwon ED, Drake CG, Scher HI, et al. Ipilimumab versus placebo after radiotherapy in patients with metastatic castration-resistant prostate cancer that had progressed after docetaxel chemotherapy (CA184-043): a multicentre, randomised, double-blind, phase 3 trial. *Lancet Oncol*. Jun 2014;15(7):700-12. doi:10.1016/s1470-2045(14)70189-5

184. Attal M, Richardson PG, Rajkumar SV, et al. Isatuximab plus pomalidomide and low-dose dexamethasone versus pomalidomide and low-dose dexamethasone in patients with relapsed and refractory multiple myeloma (ICARIA-MM): a randomised, multicentre, open-label, phase 3 study. *Lancet*. Dec 7 2019;394(10214):2096-2107. doi:10.1016/s0140-6736(19)32556-5

185. Thomas ES, Gomez HL, Li RK, et al. Ixabepilone plus capecitabine for metastatic breast cancer progressing after anthracycline and taxane treatment. *J Clin Oncol*. Nov 20 2007;25(33):5210-7. doi:10.1200/jco.2007.12.6557

186. Caplin ME, Pavel M, Ćwikła JB, et al. Lanreotide in metastatic enteropancreatic neuroendocrine tumors. *N Engl J Med*. Jul 17 2014;371(3):224-33. doi:10.1056/NEJMoa1316158

187. Hecht JR, Bang YJ, Qin SK, et al. Lapatinib in Combination With Capecitabine Plus Oxaliplatin in Human Epidermal Growth Factor Receptor 2-Positive Advanced or Metastatic Gastric, Esophageal, or Gastroesophageal Adenocarcinoma: TRIO-013/LOGiC--A Randomized Phase III Trial. *J Clin Oncol*. Feb 10 2016;34(5):443-51. doi:10.1200/jco.2015.62.6598

188. Schwartzberg LS, Franco SX, Florance A, O'Rourke L, Maltzman J, Johnston S. Lapatinib plus letrozole as first-line therapy for HER-2+ hormone receptor-positive

metastatic breast cancer. *Oncologist*. 2010;15(2):122-9. doi:10.1634/theoncologist.2009-0240

189. Satoh T, Xu RH, Chung HC, et al. Lapatinib plus paclitaxel versus paclitaxel alone in the second-line treatment of HER2-amplified advanced gastric cancer in Asian populations: TyTAN--a randomized, phase III study. *J Clin Oncol*. Jul 1 2014;32(19):2039-49. doi:10.1200/jco.2013.53.6136

190. McCarthy PL, Owzar K, Hofmeister CC, et al. Lenalidomide after stem-cell transplantation for multiple myeloma. *N Engl J Med*. May 10 2012;366(19):1770-81. doi:10.1056/NEJMoa1114083

191. Weber DM, Chen C, Niesvizky R, et al. Lenalidomide plus dexamethasone for relapsed multiple myeloma in North America. *N Engl J Med*. Nov 22 2007;357(21):2133-42. doi:10.1056/NEJMoa070596

192. Dimopoulos M, Spencer A, Attal M, et al. Lenalidomide plus dexamethasone for relapsed or refractory multiple myeloma. *N Engl J Med*. Nov 22 2007;357(21):2123-32. doi:10.1056/NEJMoa070594

193. Kudo M, Finn RS, Qin S, et al. Lenvatinib versus sorafenib in first-line treatment of patients with unresectable hepatocellular carcinoma: a randomised phase 3 non-inferiority trial. *Lancet*. Mar 24 2018;391(10126):1163-1173. doi:10.1016/s0140-6736(18)30207-1

194. Swain SM, Jeong JH, Geyer CE, Jr., et al. Longer therapy, iatrogenic amenorrhea, and survival in early breast cancer. *N Engl J Med*. Jun 3 2010;362(22):2053-65. doi:10.1056/NEJMoa0909638

195. Colleoni M, Gray KP, Gelber S, et al. Low-Dose Oral Cyclophosphamide and Methotrexate Maintenance for Hormone Receptor-Negative Early Breast Cancer: International Breast Cancer Study Group Trial 22-00. *J Clin Oncol*. Oct 1 2016;34(28):3400-8. doi:10.1200/jco.2015.65.6595

196. Dreno B, Thompson JF, Smithers BM, et al. MAGE-A3 immunotherapeutic as adjuvant therapy for patients with resected, MAGE-A3-positive, stage III melanoma (DERMA): a double-blind, randomised, placebo-controlled, phase 3 trial. *Lancet Oncol*. Jul 2018;19(7):916-929. doi:10.1016/s1470-2045(18)30254-7

© 2024 Sherry AD et al. *JAMA Network Open.*

197. Gligorov J, Doval D, Bines J, et al. Maintenance capecitabine and bevacizumab versus bevacizumab alone after initial first-line bevacizumab and docetaxel for patients with HER2-negative metastatic breast cancer (IMELDA): a randomised, open-label, phase 3 trial. *Lancet Oncol*. Nov 2014;15(12):1351-60. doi:10.1016/s1470-2045(14)70444-9

198. Golan T, Hammel P, Reni M, et al. Maintenance Olaparib for Germline BRCA-Mutated Metastatic Pancreatic Cancer. *N Engl J Med*. Jul 25 2019;381(4):317-327. doi:10.1056/NEJMoa1903387

199. Moore K, Colombo N, Scambia G, et al. Maintenance Olaparib in Patients with Newly Diagnosed Advanced Ovarian Cancer. *N Engl J Med*. Dec 27 2018;379(26):2495-2505. doi:10.1056/NEJMoa1810858

200. Paz-Ares L, de Marinis F, Dediu M, et al. Maintenance therapy with pemetrexed plus best supportive care versus placebo plus best supportive care after induction therapy with pemetrexed plus cisplatin for advanced non-squamous non-small-cell lung cancer (PARAMOUNT): a double-blind, phase 3, randomised controlled trial. *Lancet Oncol*. Mar 2012;13(3):247-55. doi:10.1016/s1470-2045(12)70063-3

201. Stewart AK, Jacobus S, Fonseca R, et al. Melphalan, prednisone, and thalidomide vs melphalan, prednisone, and lenalidomide (ECOG E1A06) in untreated multiple myeloma. *Blood*. Sep 10 2015;126(11):1294-301. doi:10.1182/blood-2014-12-613927

202. Stone RM, Mandrekar SJ, Sanford BL, et al. Midostaurin plus Chemotherapy for Acute Myeloid Leukemia with a FLT3 Mutation. *N Engl J Med*. Aug 3 2017;377(5):454-464. doi:10.1056/NEJMoa1614359

203. Kim YH, Bagot M, Pinter-Brown L, et al. Mogamulizumab versus vorinostat in previously treated cutaneous T-cell lymphoma (MAVORIC): an international, open-label, randomised, controlled phase 3 trial. *Lancet Oncol*. Sep 2018;19(9):1192-1204. doi:10.1016/s1470-2045(18)30379-6

204. Sledge GW, Jr., Toi M, Neven P, et al. MONARCH 2: Abemaciclib in Combination With Fulvestrant in Women With HR+/HER2- Advanced Breast Cancer Who Had Progressed While Receiving Endocrine Therapy. *J Clin Oncol*. Sep 1 2017;35(25):2875-2884. doi:10.1200/jco.2017.73.7585

© 2024 Sherry AD et al. *JAMA Network Open.*

205. Goetz MP, Toi M, Campone M, et al. MONARCH 3: Abemaciclib As Initial Therapy for Advanced Breast Cancer. *J Clin Oncol*. Nov 10 2017;35(32):3638-3646. doi:10.1200/jco.2017.75.6155

206. Paz-Ares L, Hirsh V, Zhang L, et al. Monotherapy Administration of Sorafenib in Patients With Non-Small Cell Lung Cancer (MISSION) Trial: A Phase III, Multicenter, Placebo-Controlled Trial of Sorafenib in Patients with Relapsed or Refractory Predominantly Nonsquamous Non-Small-Cell Lung Cancer after 2 or 3 Previous Treatment Regimens. *J Thorac Oncol*. Dec 2015;10(12):1745-53. doi:10.1097/jto.0000000000000693

207. Ajani JA, Rodriguez W, Bodoky G, et al. Multicenter phase III comparison of cisplatin/S-1 with cisplatin/infusional fluorouracil in advanced gastric or gastroesophageal adenocarcinoma study: the FLAGS trial. *J Clin Oncol*. Mar 20 2010;28(9):1547-53. doi:10.1200/jco.2009.25.4706

208. Kantarjian HM, Thomas XG, Dmoszynska A, et al. Multicenter, randomized, open-label, phase III trial of decitabine versus patient choice, with physician advice, of either supportive care or low-dose cytarabine for the treatment of older patients with newly diagnosed acute myeloid leukemia. *J Clin Oncol*. Jul 20 2012;30(21):2670-7. doi:10.1200/jco.2011.38.9429

209. Wang-Gillam A, Li CP, Bodoky G, et al. Nanoliposomal irinotecan with fluorouracil and folinic acid in metastatic pancreatic cancer after previous gemcitabine-based therapy (NAPOLI-1): a global, randomised, open-label, phase 3 trial. *Lancet*. Feb 6 2016;387(10018):545-557. doi:10.1016/s0140-6736(15)00986-1

210. Thatcher N, Hirsch FR, Luft AV, et al. Necitumumab plus gemcitabine and cisplatin versus gemcitabine and cisplatin alone as first-line therapy in patients with stage IV squamous non-small-cell lung cancer (SQUIRE): an open-label, randomised, controlled phase 3 trial. *Lancet Oncol*. Jul 2015;16(7):763-74. doi:10.1016/s1470-2045(15)00021-2

211. Gianni L, Eiermann W, Semiglazov V, et al. Neoadjuvant chemotherapy with trastuzumab followed by adjuvant trastuzumab versus neoadjuvant chemotherapy alone, in patients with HER2-positive locally advanced breast cancer (the NOAH trial): a randomised controlled superiority trial with a parallel HER2-negative cohort. *Lancet*. Jan 30 2010;375(9712):377-84. doi:10.1016/s0140-6736(09)61964-4

212. Bear HD, Tang G, Rastogi P, et al. Neoadjuvant plus adjuvant bevacizumab in early breast cancer (NSABP B-40 [NRG Oncology]): secondary outcomes of a phase 3,

randomised controlled trial. *Lancet Oncol*. Sep 2015;16(9):1037-1048. doi:10.1016/s1470-2045(15)00041-8

213. Hurvitz SA, Martin M, Symmans WF, et al. Neoadjuvant trastuzumab, pertuzumab, and chemotherapy versus trastuzumab emtansine plus pertuzumab in patients with HER2-positive breast cancer (KRISTINE): a randomised, open-label, multicentre, phase 3 trial. *Lancet Oncol*. Jan 2018;19(1):115-126. doi:10.1016/s1470-2045(17)30716-7

214. Chan A, Delaloge S, Holmes FA, et al. Neratinib after trastuzumab-based adjuvant therapy in patients with HER2-positive breast cancer (ExteNET): a multicentre, randomised, double-blind, placebo-controlled, phase 3 trial. *Lancet Oncol*. Mar 2016;17(3):367-377. doi:10.1016/s1470-2045(15)00551-3

215. Gregorc V, Gaafar RM, Favaretto A, et al. NGR-hTNF in combination with best investigator choice in previously treated malignant pleural mesothelioma (NGR015): a randomised, double-blind, placebo-controlled phase 3 trial. *Lancet Oncol*. Jun 2018;19(6):799-811. doi:10.1016/s1470-2045(18)30193-1

216. Van Cutsem E, Yoshino T, Lenz HJ, et al. Nintedanib for the treatment of patients with refractory metastatic colorectal cancer (LUME-Colon 1): a phase III, international, randomized, placebo-controlled study. *Ann Oncol*. Sep 1 2018;29(9):1955-1963. doi:10.1093/annonc/mdy241

217. Hanna NH, Kaiser R, Sullivan RN, et al. Nintedanib plus pemetrexed versus placebo plus pemetrexed in patients with relapsed or refractory, advanced non-small cell lung cancer (LUME-Lung 2): A randomized, double-blind, phase III trial. *Lung Cancer*. Dec 2016;102:65-73. doi:10.1016/j.lungcan.2016.10.011

218. Mirza MR, Monk BJ, Herrstedt J, et al. Niraparib Maintenance Therapy in Platinum-Sensitive, Recurrent Ovarian Cancer. *N Engl J Med*. Dec 1 2016;375(22):2154-2164. doi:10.1056/NEJMoa1611310

219. Ferris RL, Blumenschein G, Jr., Fayette J, et al. Nivolumab for Recurrent Squamous-Cell Carcinoma of the Head and Neck. *N Engl J Med*. Nov 10 2016;375(19):1856-1867. doi:10.1056/NEJMoa1602252

220. Motzer RJ, Tannir NM, McDermott DF, et al. Nivolumab plus Ipilimumab versus Sunitinib in Advanced Renal-Cell Carcinoma. *N Engl J Med*. Apr 5 2018;378(14):1277-1290. doi:10.1056/NEJMoa1712126

© 2024 Sherry AD et al. *JAMA Network Open.*

221. Weber JS, D'Angelo SP, Minor D, et al. Nivolumab versus chemotherapy in patients with advanced melanoma who progressed after anti-CTLA-4 treatment (CheckMate 037): a randomised, controlled, open-label, phase 3 trial. *Lancet Oncol*. Apr 2015;16(4):375-84. doi:10.1016/s1470-2045(15)70076-8

222. Borghaei H, Paz-Ares L, Horn L, et al. Nivolumab versus Docetaxel in Advanced Nonsquamous Non-Small-Cell Lung Cancer. *N Engl J Med*. Oct 22 2015;373(17):1627-39. doi:10.1056/NEJMoa1507643

223. Brahmer J, Reckamp KL, Baas P, et al. Nivolumab versus Docetaxel in Advanced Squamous-Cell Non-Small-Cell Lung Cancer. *N Engl J Med*. Jul 9 2015;373(2):123-35. doi:10.1056/NEJMoa1504627

224. Motzer RJ, Escudier B, McDermott DF, et al. Nivolumab versus Everolimus in Advanced Renal-Cell Carcinoma. *N Engl J Med*. Nov 5 2015;373(19):1803-13. doi:10.1056/NEJMoa1510665

225. Vitolo U, Trněný M, Belada D, et al. Obinutuzumab or Rituximab Plus Cyclophosphamide, Doxorubicin, Vincristine, and Prednisone in Previously Untreated Diffuse Large B-Cell Lymphoma. *J Clin Oncol*. Nov 1 2017;35(31):3529-3537. doi:10.1200/jco.2017.73.3402

226. Sehn LH, Chua N, Mayer J, et al. Obinutuzumab plus bendamustine versus bendamustine monotherapy in patients with rituximab-refractory indolent non-Hodgkin lymphoma (GADOLIN): a randomised, controlled, open-label, multicentre, phase 3 trial. *Lancet Oncol*. Aug 2016;17(8):1081-1093. doi:10.1016/s1470-2045(16)30097-3

227. Aghajanian C, Blank SV, Goff BA, et al. OCEANS: a randomized, double-blind, placebo-controlled phase III trial of chemotherapy with or without bevacizumab in patients with platinum-sensitive recurrent epithelial ovarian, primary peritoneal, or fallopian tube cancer. *J Clin Oncol*. Jun 10 2012;30(17):2039-45. doi:10.1200/jco.2012.42.0505

228. van Oers MH, Kuliczkowski K, Smolej L, et al. Ofatumumab maintenance versus observation in relapsed chronic lymphocytic leukaemia (PROLONG): an open-label, multicentre, randomised phase 3 study. *Lancet Oncol*. Oct 2015;16(13):1370-9. doi:10.1016/s1470-2045(15)00143-6

229. Robak T, Warzocha K, Govind Babu K, et al. Ofatumumab plus fludarabine and cyclophosphamide in relapsed chronic lymphocytic leukemia: results from the

© 2024 Sherry AD et al. *JAMA Network Open.*

COMPLEMENT 2 trial. *Leuk Lymphoma*. May 2017;58(5):1084-1093. doi:10.1080/10428194.2016.1233536

230. Robson M, Im SA, Senkus E, et al. Olaparib for Metastatic Breast Cancer in Patients with a Germline BRCA Mutation. *N Engl J Med*. Aug 10 2017;377(6):523-533. doi:10.1056/NEJMoa1706450

231. Bang YJ, Xu RH, Chin K, et al. Olaparib in combination with paclitaxel in patients with advanced gastric cancer who have progressed following first-line therapy (GOLD): a double-blind, randomised, placebo-controlled, phase 3 trial. *Lancet Oncol*. Dec 2017;18(12):1637-1651. doi:10.1016/s1470-2045(17)30682-4

232. Moreau P, Mateos MV, Berenson JR, et al. Once weekly versus twice weekly carfilzomib dosing in patients with relapsed and refractory multiple myeloma (A.R.R.O.W.): interim analysis results of a randomised, phase 3 study. *Lancet Oncol*. Jul 2018;19(7):953-964. doi:10.1016/s1470-2045(18)30354-1

233. Van Cutsem E, Peeters M, Siena S, et al. Open-label phase III trial of panitumumab plus best supportive care compared with best supportive care alone in patients with chemotherapy-refractory metastatic colorectal cancer. *J Clin Oncol*. May 1 2007;25(13):1658-64. doi:10.1200/jco.2006.08.1620

234. Dimopoulos MA, Gay F, Schjesvold F, et al. Oral ixazomib maintenance following autologous stem cell transplantation (TOURMALINE-MM3): a double-blind, randomised, placebo-controlled phase 3 trial. *Lancet*. Jan 19 2019;393(10168):253-264. doi:10.1016/s0140-6736(18)33003-4

235. Moreau P, Masszi T, Grzasko N, et al. Oral Ixazomib, Lenalidomide, and Dexamethasone for Multiple Myeloma. *N Engl J Med*. Apr 28 2016;374(17):1621-34. doi:10.1056/NEJMoa1516282

236. Kudo M, Cheng AL, Park JW, et al. Orantinib versus placebo combined with transcatheter arterial chemoembolisation in patients with unresectable hepatocellular carcinoma (ORIENTAL): a randomised, double-blind, placebo-controlled, multicentre, phase 3 study. *Lancet Gastroenterol Hepatol*. Jan 2018;3(1):37-46. doi:10.1016/s2468-1253(17)30290-x

237. Saad F, Fizazi K, Jinga V, et al. Orteronel plus prednisone in patients with chemotherapy-naive metastatic castration-resistant prostate cancer (ELM-PC 4): a double-blind, multicentre, phase 3, randomised, placebo-controlled trial. *Lancet Oncol*. Mar 2015;16(3):338-48. doi:10.1016/s1470-2045(15)70027-6

© 2024 Sherry AD et al. *JAMA Network Open.*

238. Soria JC, Ohe Y, Vansteenkiste J, et al. Osimertinib in Untreated EGFR-Mutated Advanced Non-Small-Cell Lung Cancer. *N Engl J Med*. Jan 11 2018;378(2):113-125. doi:10.1056/NEJMoa1713137

239. Mok TS, Wu YL, Ahn MJ, et al. Osimertinib or Platinum-Pemetrexed in EGFR T790M-Positive Lung Cancer. *N Engl J Med*. Feb 16 2017;376(7):629-640. doi:10.1056/NEJMoa1612674

240. Finn RS, Martin M, Rugo HS, et al. Palbociclib and Letrozole in Advanced Breast Cancer. *N Engl J Med*. Nov 17 2016;375(20):1925-1936. doi:10.1056/NEJMoa1607303

241. Turner NC, Ro J, André F, et al. Palbociclib in Hormone-Receptor-Positive Advanced Breast Cancer. *N Engl J Med*. Jul 16 2015;373(3):209-19. doi:10.1056/NEJMoa1505270

242. Price TJ, Peeters M, Kim TW, et al. Panitumumab versus cetuximab in patients with chemotherapy-refractory wild-type KRAS exon 2 metastatic colorectal cancer (ASPECCT): a randomised, multicentre, open-label, non-inferiority phase 3 study. *Lancet Oncol*. May 2014;15(6):569-79. doi:10.1016/s1470-2045(14)70118-4

243. San-Miguel JF, Hungria VT, Yoon SS, et al. Panobinostat plus bortezomib and dexamethasone versus placebo plus bortezomib and dexamethasone in patients with relapsed or relapsed and refractory multiple myeloma: a multicentre, randomised, double-blind phase 3 trial. *Lancet Oncol*. Oct 2014;15(11):1195-206. doi:10.1016/s1470-2045(14)70440-1

244. Sternberg CN, Davis ID, Mardiak J, et al. Pazopanib in locally advanced or metastatic renal cell carcinoma: results of a randomized phase III trial. *J Clin Oncol*. Feb 20 2010;28(6):1061-8. doi:10.1200/jco.2009.23.9764

245. Bellmunt J, de Wit R, Vaughn DJ, et al. Pembrolizumab as Second-Line Therapy for Advanced Urothelial Carcinoma. *N Engl J Med*. Mar 16 2017;376(11):1015-1026. doi:10.1056/NEJMoa1613683

246. Rini BI, Plimack ER, Stus V, et al. Pembrolizumab plus Axitinib versus Sunitinib for Advanced Renal-Cell Carcinoma. *N Engl J Med*. Mar 21 2019;380(12):1116-1127. doi:10.1056/NEJMoa1816714

© 2024 Sherry AD et al. *JAMA Network Open.*

247. Paz-Ares L, Luft A, Vicente D, et al. Pembrolizumab plus Chemotherapy for Squamous Non-Small-Cell Lung Cancer. *N Engl J Med*. Nov 22 2018;379(21):2040-2051. doi:10.1056/NEJMoa1810865

248. Gandhi L, Rodríguez-Abreu D, Gadgeel S, et al. Pembrolizumab plus Chemotherapy in Metastatic Non-Small-Cell Lung Cancer. *N Engl J Med*. May 31 2018;378(22):2078-2092. doi:10.1056/NEJMoa1801005

249. Reck M, Rodríguez-Abreu D, Robinson AG, et al. Pembrolizumab versus Chemotherapy for PD-L1-Positive Non-Small-Cell Lung Cancer. *N Engl J Med*. Nov 10 2016;375(19):1823-1833. doi:10.1056/NEJMoa1606774

250. Herbst RS, Baas P, Kim DW, et al. Pembrolizumab versus docetaxel for previously treated, PD-L1-positive, advanced non-small-cell lung cancer (KEYNOTE-010): a randomised controlled trial. *Lancet*. Apr 9 2016;387(10027):1540-1550. doi:10.1016/s0140-6736(15)01281-7

251. Robert C, Schachter J, Long GV, et al. Pembrolizumab versus Ipilimumab in Advanced Melanoma. *N Engl J Med*. Jun 25 2015;372(26):2521-32. doi:10.1056/NEJMoa1503093

252. Cohen EEW, Soulières D, Le Tourneau C, et al. Pembrolizumab versus methotrexate, docetaxel, or cetuximab for recurrent or metastatic head-and-neck squamous cell carcinoma (KEYNOTE-040): a randomised, open-label, phase 3 study. *Lancet*. Jan 12 2019;393(10167):156-167. doi:10.1016/s0140-6736(18)31999-8

253. Shitara K, Özgüroğlu M, Bang YJ, et al. Pembrolizumab versus paclitaxel for previously treated, advanced gastric or gastro-oesophageal junction cancer (KEYNOTE-061): a randomised, open-label, controlled, phase 3 trial. *Lancet*. Jul 14 2018;392(10142):123-133. doi:10.1016/s0140-6736(18)31257-1

254. Urba S, van Herpen CM, Sahoo TP, et al. Pemetrexed in combination with cisplatin versus cisplatin monotherapy in patients with recurrent or metastatic head and neck cancer: final results of a randomized, double-blind, placebo-controlled, phase 3 study. *Cancer*. Oct 1 2012;118(19):4694-705. doi:10.1002/cncr.27449

255. Tabernero J, Hoff PM, Shen L, et al. Pertuzumab plus trastuzumab and chemotherapy for HER2-positive metastatic gastric or gastro-oesophageal junction cancer (JACOB): final analysis of a double-blind, randomised, placebo-controlled phase 3 study. *Lancet Oncol*. Oct 2018;19(10):1372-1384. doi:10.1016/s1470-2045(18)30481-9

© 2024 Sherry AD et al. *JAMA Network Open.*

256. Baselga J, Cortés J, Kim SB, et al. Pertuzumab plus trastuzumab plus docetaxel for metastatic breast cancer. *N Engl J Med*. Jan 12 2012;366(2):109-19. doi:10.1056/NEJMoa1113216

257. Flinn IW, Hillmen P, Montillo M, et al. The phase 3 DUO trial: duvelisib vs ofatumumab in relapsed and refractory CLL/SLL. *Blood*. Dec 6 2018;132(23):2446-2455. doi:10.1182/blood-2018-05-850461

258. Monk BJ, Herzog TJ, Wang G, et al. A phase 3 randomized, open-label, multicenter trial for safety and efficacy of combined trabectedin and pegylated liposomal doxorubicin therapy for recurrent ovarian cancer. *Gynecol Oncol*. Mar 2020;156(3):535-544. doi:10.1016/j.ygyno.2019.12.043

259. Horvath N, Spencer A, Kenealy M, et al. Phase 3 study of subcutaneous bortezomib, thalidomide, and prednisolone consolidation after subcutaneous bortezomib-based induction and autologous stem cell transplantation in patients with previously untreated multiple myeloma: the VCAT study. *Leuk Lymphoma*. Sep 2019;60(9):2122-2133. doi:10.1080/10428194.2019.1579322

260. Strosberg J, El-Haddad G, Wolin E, et al. Phase 3 Trial of (177)Lu-Dotatate for Midgut Neuroendocrine Tumors. *N Engl J Med*. Jan 12 2017;376(2):125-135. doi:10.1056/NEJMoa1607427

261. Nelson JB, Fizazi K, Miller K, et al. Phase 3, randomized, placebo-controlled study of zibotentan (ZD4054) in patients with castration-resistant prostate cancer metastatic to bone. *Cancer*. Nov 15 2012;118(22):5709-18. doi:10.1002/cncr.27674

262. Tak WY, Lin SM, Wang Y, et al. Phase III HEAT Study Adding Lyso-Thermosensitive Liposomal Doxorubicin to Radiofrequency Ablation in Patients with Unresectable Hepatocellular Carcinoma Lesions. *Clin Cancer Res*. Jan 1 2018;24(1):73-83. doi:10.1158/1078-0432.Ccr-16-2433

263. Agarwala SS, Lee SJ, Yip W, et al. Phase III Randomized Study of 4 Weeks of High-Dose Interferon-α-2b in Stage T2bNO, T3a-bNO, T4a-bNO, and T1-4N1a-2a (microscopic) Melanoma: A Trial of the Eastern Cooperative Oncology Group-American College of Radiology Imaging Network Cancer Research Group (E1697). *J Clin Oncol*. Mar 10 2017;35(8):885-892. doi:10.1200/jco.2016.70.2951

264. Slamon DJ, Neven P, Chia S, et al. Phase III Randomized Study of Ribociclib and Fulvestrant in Hormone Receptor-Positive, Human Epidermal Growth Factor

© 2024 Sherry AD et al. *JAMA Network Open.*

Receptor 2-Negative Advanced Breast Cancer: MONALEESA-3. *J Clin Oncol*. Aug 20 2018;36(24):2465-2472. doi:10.1200/jco.2018.78.9909

265. Reck M, Luft A, Szczesna A, et al. Phase III Randomized Trial of Ipilimumab Plus Etoposide and Platinum Versus Placebo Plus Etoposide and Platinum in Extensive-Stage Small-Cell Lung Cancer. *J Clin Oncol*. Nov 1 2016;34(31):3740-3748. doi:10.1200/jco.2016.67.6601

266. Argiris A, Ghebremichael M, Gilbert J, et al. Phase III randomized, placebo-controlled trial of docetaxel with or without gefitinib in recurrent or metastatic head and neck cancer: an eastern cooperative oncology group trial. *J Clin Oncol*. Apr 10 2013;31(11):1405-14. doi:10.1200/jco.2012.45.4272

267. Eisenberger M, Hardy-Bessard AC, Kim CS, et al. Phase III Study Comparing a Reduced Dose of Cabazitaxel (20 mg/m(2)) and the Currently Approved Dose (25 mg/m(2)) in Postdocetaxel Patients With Metastatic Castration-Resistant Prostate Cancer-PROSELICA. *J Clin Oncol*. Oct 1 2017;35(28):3198-3206. doi:10.1200/jco.2016.72.1076

268. Sequist LV, Yang JC, Yamamoto N, et al. Phase III study of afatinib or cisplatin plus pemetrexed in patients with metastatic lung adenocarcinoma with EGFR mutations. *J Clin Oncol*. Sep 20 2013;31(27):3327-34. doi:10.1200/jco.2012.44.2806

269. Miles DW, Chan A, Dirix LY, et al. Phase III study of bevacizumab plus docetaxel compared with placebo plus docetaxel for the first-line treatment of human epidermal growth factor receptor 2-negative metastatic breast cancer. *J Clin Oncol*. Jul 10 2010;28(20):3239-47. doi:10.1200/jco.2008.21.6457

270. Scagliotti G, Novello S, von Pawel J, et al. Phase III study of carboplatin and paclitaxel alone or with sorafenib in advanced non-small-cell lung cancer. *J Clin Oncol*. Apr 10 2010;28(11):1835-42. doi:10.1200/jco.2009.26.1321

271. Kudo M, Imanaka K, Chida N, et al. Phase III study of sorafenib after transarterial chemoembolisation in Japanese and Korean patients with unresectable hepatocellular carcinoma. *Eur J Cancer*. Sep 2011;47(14):2117-27. doi:10.1016/j.ejca.2011.05.007

272. Dickler MN, Barry WT, Cirrincione CT, et al. Phase III Trial Evaluating Letrozole As First-Line Endocrine Therapy With or Without Bevacizumab for the Treatment of Postmenopausal Women With Hormone Receptor-Positive Advanced-Stage Breast Cancer: CALGB 40503 (Alliance). *J Clin Oncol*. Aug 1 2016;34(22):2602-9. doi:10.1200/jco.2015.66.1595

© 2024 Sherry AD et al. *JAMA Network Open.*

273. Govindan R, Szczesna A, Ahn MJ, et al. Phase III Trial of Ipilimumab Combined With Paclitaxel and Carboplatin in Advanced Squamous Non-Small-Cell Lung Cancer. *J Clin Oncol*. Oct 20 2017;35(30):3449-3457. doi:10.1200/jco.2016.71.7629

274. Crown JP, Diéras V, Staroslawska E, et al. Phase III trial of sunitinib in combination with capecitabine versus capecitabine monotherapy for the treatment of patients with pretreated metastatic breast cancer. *J Clin Oncol*. Aug 10 2013;31(23):2870-8. doi:10.1200/jco.2012.43.3391

275. Natale RB, Thongprasert S, Greco FA, et al. Phase III trial of vandetanib compared with erlotinib in patients with previously treated advanced non-small-cell lung cancer. *J Clin Oncol*. Mar 10 2011;29(8):1059-66. doi:10.1200/jco.2010.28.5981

276. Johnston SRD, Hegg R, Im SA, et al. Phase III, Randomized Study of Dual Human Epidermal Growth Factor Receptor 2 (HER2) Blockade With Lapatinib Plus Trastuzumab in Combination With an Aromatase Inhibitor in Postmenopausal Women With HER2-Positive, Hormone Receptor-Positive Metastatic Breast Cancer: ALTERNATIVE. *J Clin Oncol*. Mar 10 2018;36(8):741-748. doi:10.1200/jco.2017.74.7824

277. Österborg A, Udvardy M, Zaritskey A, et al. Phase III, randomized study of ofatumumab versus physicians' choice of therapy and standard versus extended-length ofatumumab in patients with bulky fludarabine-refractory chronic lymphocytic leukemia. *Leuk Lymphoma*. Sep 2016;57(9):2037-46. doi:10.3109/10428194.2015.1122783

278. Fizazi K, Jones R, Oudard S, et al. Phase III, randomized, double-blind, multicenter trial comparing orteronel (TAK-700) plus prednisone with placebo plus prednisone in patients with metastatic castration-resistant prostate cancer that has progressed during or after docetaxel-based therapy: ELM-PC 5. *J Clin Oncol*. Mar 1 2015;33(7):723-31. doi:10.1200/jco.2014.56.5119

279. Pinter T, Klippel Z, Cesas A, et al. A Phase III, Randomized, Double-Blind, Placebo-Controlled Trial of Pegfilgrastim in Patients Receiving First-Line FOLFOX/Bevacizumab or FOLFIRI/Bevacizumab for Locally Advanced or Metastatic Colorectal Cancer: Final Results of the Pegfilgrastim and Anti-VEGF Evaluation Study (PAVES). *Clin Colorectal Cancer*. Jun 2017;16(2):103-114.e3. doi:10.1016/j.clcc.2016.08.008

280. Fizazi K, Higano CS, Nelson JB, et al. Phase III, randomized, placebo-controlled study of docetaxel in combination with zibotentan in patients with metastatic castration-

© 2024 Sherry AD et al. *JAMA Network Open.*

resistant prostate cancer. *J Clin Oncol*. May 10 2013;31(14):1740-7. doi:10.1200/jco.2012.46.4149

281. Pettengell R, Coiffier B, Narayanan G, et al. Pixantrone dimaleate versus other chemotherapeutic agents as a single-agent salvage treatment in patients with relapsed or refractory aggressive non-Hodgkin lymphoma: a phase 3, multicentre, open-label, randomised trial. *Lancet Oncol*. Jul 2012;13(7):696-706. doi:10.1016/s1470-2045(12)70212-7

282. Rinke A, Müller HH, Schade-Brittinger C, et al. Placebo-controlled, double-blind, prospective, randomized study on the effect of octreotide LAR in the control of tumor growth in patients with metastatic neuroendocrine midgut tumors: a report from the PROMID Study Group. *J Clin Oncol*. Oct 1 2009;27(28):4656-63. doi:10.1200/jco.2009.22.8510

283. Vermorken JB, Mesia R, Rivera F, et al. Platinum-based chemotherapy plus cetuximab in head and neck cancer. *N Engl J Med*. Sep 11 2008;359(11):1116-27. doi:10.1056/NEJMoa0802656

284. Patel JD, Socinski MA, Garon EB, et al. PointBreak: a randomized phase III study of pemetrexed plus carboplatin and bevacizumab followed by maintenance pemetrexed and bevacizumab versus paclitaxel plus carboplatin and bevacizumab followed by maintenance bevacizumab in patients with stage IIIB or IV nonsquamous non-small-cell lung cancer. *J Clin Oncol*. Dec 1 2013;31(34):4349-57. doi:10.1200/jco.2012.47.9626

285. Miguel JS, Weisel K, Moreau P, et al. Pomalidomide plus low-dose dexamethasone versus high-dose dexamethasone alone for patients with relapsed and refractory multiple myeloma (MM-003): a randomised, open-label, phase 3 trial. *Lancet Oncol*. Oct 2013;14(11):1055-1066. doi:10.1016/s1470-2045(13)70380-2

286. Seidman AD, Chan S, Wang J, Zhu C, Xu C, Xu B. A pooled analysis of gemcitabine plus docetaxel versus capecitabine plus docetaxel in metastatic breast cancer. *Oncologist*. May 2014;19(5):443-52. doi:10.1634/theoncologist.2013-0428

287. Harrington K, Temam S, Mehanna H, et al. Postoperative Adjuvant Lapatinib and Concurrent Chemoradiotherapy Followed by Maintenance Lapatinib Monotherapy in High-Risk Patients With Resected Squamous Cell Carcinoma of the Head and Neck: A Phase III, Randomized, Double-Blind, Placebo-Controlled Study. *J Clin Oncol*. Dec 10 2015;33(35):4202-9. doi:10.1200/jco.2015.61.4370

© 2024 Sherry AD et al. *JAMA Network Open.*

288. de Bono JS, Oudard S, Ozguroglu M, et al. Prednisone plus cabazitaxel or mitoxantrone for metastatic castration-resistant prostate cancer progressing after docetaxel treatment: a randomised open-label trial. *Lancet*. Oct 2 2010;376(9747):1147-54. doi:10.1016/s0140-6736(10)61389-x

289. Mackey JR, Ramos-Vazquez M, Lipatov O, et al. Primary results of ROSE/TRIO-12, a randomized placebo-controlled phase III trial evaluating the addition of ramucirumab to first-line docetaxel chemotherapy in metastatic breast cancer. *J Clin Oncol*. Jan 10 2015;33(2):141-8. doi:10.1200/jco.2014.57.1513

290. Senan S, Brade A, Wang LH, et al. PROCLAIM: Randomized Phase III Trial of Pemetrexed-Cisplatin or Etoposide-Cisplatin Plus Thoracic Radiation Therapy Followed by Consolidation Chemotherapy in Locally Advanced Nonsquamous Non-Small-Cell Lung Cancer. *J Clin Oncol*. Mar 20 2016;34(9):953-62. doi:10.1200/jco.2015.64.8824

291. Zinner RG, Obasaju CK, Spigel DR, et al. PRONOUNCE: randomized, open-label, phase III study of first-line pemetrexed + carboplatin followed by maintenance pemetrexed versus paclitaxel + carboplatin + bevacizumab followed by maintenance bevacizumab in patients ith advanced nonsquamous non-small-cell lung cancer. *J Thorac Oncol*. Jan 2015;10(1):134-42. doi:10.1097/jto.0000000000000366

292. Cortes JE, Khaled S, Martinelli G, et al. Quizartinib versus salvage chemotherapy in relapsed or refractory FLT3-ITD acute myeloid leukaemia (QuANTUM-R): a multicentre, randomised, controlled, open-label, phase 3 trial. *Lancet Oncol*. Jul 2019;20(7):984-997. doi:10.1016/s1470-2045(19)30150-0

293. Shipley WU, Seiferheld W, Lukka HR, et al. Radiation with or without Antiandrogen Therapy in Recurrent Prostate Cancer. *N Engl J Med*. Feb 2 2017;376(5):417-428. doi:10.1056/NEJMoa1607529

294. Wilt TJ, Brawer MK, Jones KM, et al. Radical prostatectomy versus observation for localized prostate cancer. *N Engl J Med*. Jul 19 2012;367(3):203-13. doi:10.1056/NEJMoa1113162

295. Jones CU, Hunt D, McGowan DG, et al. Radiotherapy and short-term androgen deprivation for localized prostate cancer. *N Engl J Med*. Jul 14 2011;365(2):107-18. doi:10.1056/NEJMoa1012348

296. Gillison ML, Trotti AM, Harris J, et al. Radiotherapy plus cetuximab or cisplatin in human papillomavirus-positive oropharyngeal cancer (NRG Oncology RTOG 1016): a

© 2024 Sherry AD et al. *JAMA Network Open.*

randomised, multicentre, non-inferiority trial. *Lancet*. Jan 5 2019;393(10166):40-50. doi:10.1016/s0140-6736(18)32779-x

297. Fuchs CS, Tomasek J, Yong CJ, et al. Ramucirumab monotherapy for previously treated advanced gastric or gastro-oesophageal junction adenocarcinoma (REGARD): an international, randomised, multicentre, placebo-controlled, phase 3 trial. *Lancet*. Jan 4 2014;383(9911):31-39. doi:10.1016/s0140-6736(13)61719-5

298. Petrylak DP, de Wit R, Chi KN, et al. Ramucirumab plus docetaxel versus placebo plus docetaxel in patients with locally advanced or metastatic urothelial carcinoma after platinum-based therapy (RANGE): a randomised, double-blind, phase 3 trial. *Lancet*. Nov 18 2017;390(10109):2266-2277. doi:10.1016/s0140-6736(17)32365-6

299. Wilke H, Muro K, Van Cutsem E, et al. Ramucirumab plus paclitaxel versus placebo plus paclitaxel in patients with previously treated advanced gastric or gastro-oesophageal junction adenocarcinoma (RAINBOW): a double-blind, randomised phase 3 trial. *Lancet Oncol*. Oct 2014;15(11):1224-35. doi:10.1016/s1470-2045(14)70420-6

300. Zhu AX, Park JO, Ryoo BY, et al. Ramucirumab versus placebo as second-line treatment in patients with advanced hepatocellular carcinoma following first-line therapy with sorafenib (REACH): a randomised, double-blind, multicentre, phase 3 trial. *Lancet Oncol*. Jul 2015;16(7):859-70. doi:10.1016/s1470-2045(15)00050-9

301. Tabernero J, Yoshino T, Cohn AL, et al. Ramucirumab versus placebo in combination with second-line FOLFIRI in patients with metastatic colorectal carcinoma that progressed during or after first-line therapy with bevacizumab, oxaliplatin, and a fluoropyrimidine (RAISE): a randomised, double-blind, multicentre, phase 3 study. *Lancet Oncol*. May 2015;16(5):499-508. doi:10.1016/s1470-2045(15)70127-0

302. Fuchs CS, Shitara K, Di Bartolomeo M, et al. Ramucirumab with cisplatin and fluoropyrimidine as first-line therapy in patients with metastatic gastric or junctional adenocarcinoma (RAINFALL): a double-blind, randomised, placebo-controlled, phase 3 trial. *Lancet Oncol*. Mar 2019;20(3):420-435. doi:10.1016/s1470-2045(18)30791-5

303. Rougier P, Riess H, Manges R, et al. Randomised, placebo-controlled, double-blind, parallel-group phase III study evaluating aflibercept in patients receiving first-line treatment with gemcitabine for metastatic pancreatic cancer. *Eur J Cancer*. Aug 2013;49(12):2633-42. doi:10.1016/j.ejca.2013.04.002

304. Smith MR, Halabi S, Ryan CJ, et al. Randomized controlled trial of early zoledronic acid in men with castration-sensitive prostate cancer and bone metastases:

results of CALGB 90202 (alliance). *J Clin Oncol*. Apr 10 2014;32(11):1143-50. doi:10.1200/jco.2013.51.6500

305. Fenaux P, Giagounidis A, Selleslag D, et al. A randomized phase 3 study of lenalidomide versus placebo in RBC transfusion-dependent patients with Low-/Intermediate-1-risk myelodysplastic syndromes with del5q. *Blood*. Oct 6 2011;118(14):3765-76. doi:10.1182/blood-2011-01-330126

306. Cassidy J, Clarke S, Díaz-Rubio E, et al. Randomized phase III study of capecitabine plus oxaliplatin compared with fluorouracil/folinic acid plus oxaliplatin as first-line therapy for metastatic colorectal cancer. *J Clin Oncol*. Apr 20 2008;26(12):2006-12. doi:10.1200/jco.2007.14.9898

307. Pillai RN, Fennell DA, Kovcin V, et al. Randomized Phase III Study of Ganetespib, a Heat Shock Protein 90 Inhibitor, With Docetaxel Versus Docetaxel in Advanced Non-Small-Cell Lung Cancer (GALAXY-2). *J Clin Oncol*. Feb 20 2020;38(6):613-622. doi:10.1200/jco.19.00816

308. Peeters M, Price TJ, Cervantes A, et al. Randomized phase III study of panitumumab with fluorouracil, leucovorin, and irinotecan (FOLFIRI) compared with FOLFIRI alone as second-line treatment in patients with metastatic colorectal cancer. *J Clin Oncol*. Nov 1 2010;28(31):4706-13. doi:10.1200/jco.2009.27.6055

309. Orlowski RZ, Nagler A, Sonneveld P, et al. Randomized phase III study of pegylated liposomal doxorubicin plus bortezomib compared with bortezomib alone in relapsed or refractory multiple myeloma: combination therapy improves time to progression. *J Clin Oncol*. Sep 1 2007;25(25):3892-901. doi:10.1200/jco.2006.10.5460

310. Hoang T, Dahlberg SE, Schiller JH, et al. Randomized phase III study of thoracic radiation in combination with paclitaxel and carboplatin with or without thalidomide in patients with stage III non-small-cell lung cancer: the ECOG 3598 study. *J Clin Oncol*. Feb 20 2012;30(6):616-22. doi:10.1200/jco.2011.36.9116

311. Homesley HD, Filiaci V, Gibbons SK, et al. A randomized phase III trial in advanced endometrial carcinoma of surgery and volume directed radiation followed by cisplatin and doxorubicin with or without paclitaxel: A Gynecologic Oncology Group study. *Gynecol Oncol*. Mar 2009;112(3):543-52. doi:10.1016/j.ygyno.2008.11.014

312. Ang KK, Zhang Q, Rosenthal DI, et al. Randomized phase III trial of concurrent accelerated radiation plus cisplatin with or without cetuximab for stage III to IV head and

neck carcinoma: RTOG 0522. *J Clin Oncol*. Sep 20 2014;32(27):2940-50. doi:10.1200/jco.2013.53.5633

313. Sparano JA, Vrdoljak E, Rixe O, et al. Randomized phase III trial of ixabepilone plus capecitabine versus capecitabine in patients with metastatic breast cancer previously treated with an anthracycline and a taxane. *J Clin Oncol*. Jul 10 2010;28(20):3256-63. doi:10.1200/jco.2009.24.4244

314. Barlesi F, Scherpereel A, Rittmeyer A, et al. Randomized phase III trial of maintenance bevacizumab with or without pemetrexed after first-line induction with bevacizumab, cisplatin, and pemetrexed in advanced nonsquamous non-small-cell lung cancer: AVAPERL (MO22089). *J Clin Oncol*. Aug 20 2013;31(24):3004-11. doi:10.1200/jco.2012.42.3749

315. Hutson TE, Escudier B, Esteban E, et al. Randomized phase III trial of temsirolimus versus sorafenib as second-line therapy after sunitinib in patients with metastatic renal cell carcinoma. *J Clin Oncol*. Mar 10 2014;32(8):760-7. doi:10.1200/jco.2013.50.3961

316. Urruticoechea A, Rizwanullah M, Im SA, et al. Randomized Phase III Trial of Trastuzumab Plus Capecitabine With or Without Pertuzumab in Patients With Human Epidermal Growth Factor Receptor 2-Positive Metastatic Breast Cancer Who Experienced Disease Progression During or After Trastuzumab-Based Therapy. *J Clin Oncol*. Sep 10 2017;35(26):3030-3038. doi:10.1200/jco.2016.70.6267

317. Sosa JA, Elisei R, Jarzab B, et al. Randomized safety and efficacy study of fosbretabulin with paclitaxel/carboplatin against anaplastic thyroid carcinoma. *Thyroid*. Feb 2014;24(2):232-40. doi:10.1089/thy.2013.0078

318. Blackwell KL, Burstein HJ, Storniolo AM, et al. Randomized study of Lapatinib alone or in combination with trastuzumab in women with ErbB2-positive, trastuzumab-refractory metastatic breast cancer. *J Clin Oncol*. Mar 1 2010;28(7):1124-30. doi:10.1200/jco.2008.21.4437

319. Gilbert MR, Dignam JJ, Armstrong TS, et al. A randomized trial of bevacizumab for newly diagnosed glioblastoma. *N Engl J Med*. Feb 20 2014;370(8):699-708. doi:10.1056/NEJMoa1308573

320. Coombes RC, Hall E, Gibson LJ, et al. A randomized trial of exemestane after two to three years of tamoxifen therapy in postmenopausal women with primary breast cancer. *N Engl J Med*. Mar 11 2004;350(11):1081-92. doi:10.1056/NEJMoa040331

© 2024 Sherry AD et al. *JAMA Network Open.*

321. Hersh EM, Del Vecchio M, Brown MP, et al. A randomized, controlled phase III trial of nab-Paclitaxel versus dacarbazine in chemotherapy-naïve patients with metastatic melanoma. *Ann Oncol*. Nov 2015;26(11):2267-74. doi:10.1093/annonc/mdv324

322. Smit EF, Wu YL, Gervais R, et al. A randomized, double-blind, phase III study comparing two doses of erlotinib for second-line treatment of current smokers with advanced non-small-cell lung cancer (CurrentS). *Lung Cancer*. Sep 2016;99:94-101. doi:10.1016/j.lungcan.2016.06.019

323. Beer TM, Kwon ED, Drake CG, et al. Randomized, Double-Blind, Phase III Trial of Ipilimumab Versus Placebo in Asymptomatic or Minimally Symptomatic Patients With Metastatic Chemotherapy-Naive Castration-Resistant Prostate Cancer. *J Clin Oncol*. Jan 2017;35(1):40-47. doi:10.1200/jco.2016.69.1584

324. Kelly WK, Halabi S, Carducci M, et al. Randomized, double-blind, placebo-controlled phase III trial comparing docetaxel and prednisone with or without bevacizumab in men with metastatic castration-resistant prostate cancer: CALGB 90401. *J Clin Oncol*. May 1 2012;30(13):1534-40. doi:10.1200/jco.2011.39.4767

325. Li J, Qin S, Xu J, et al. Randomized, Double-Blind, Placebo-Controlled Phase III Trial of Apatinib in Patients With Chemotherapy-Refractory Advanced or Metastatic Adenocarcinoma of the Stomach or Gastroesophageal Junction. *J Clin Oncol*. May 1 2016;34(13):1448-54. doi:10.1200/jco.2015.63.5995

326. Leyland-Jones B, Bondarenko I, Nemsadze G, et al. A Randomized, Open-Label, Multicenter, Phase III Study of Epoetin Alfa Versus Best Standard of Care in Anemic Patients With Metastatic Breast Cancer Receiving Standard Chemotherapy. *J Clin Oncol*. Apr 10 2016;34(11):1197-207. doi:10.1200/jco.2015.63.5649

327. Langer CJ, Novello S, Park K, et al. Randomized, phase III trial of first-line figitumumab in combination with paclitaxel and carboplatin versus paclitaxel and carboplatin alone in patients with advanced non-small-cell lung cancer. *J Clin Oncol*. Jul 1 2014;32(19):2059-66. doi:10.1200/jco.2013.54.4932

328. Douillard JY, Siena S, Cassidy J, et al. Randomized, phase III trial of panitumumab with infusional fluorouracil, leucovorin, and oxaliplatin (FOLFOX4) versus FOLFOX4 alone as first-line treatment in patients with previously untreated metastatic colorectal cancer: the PRIME study. *J Clin Oncol*. Nov 1 2010;28(31):4697-705. doi:10.1200/jco.2009.27.4860

© 2024 Sherry AD et al. *JAMA Network Open.*

329. Whelan TJ, Olivotto IA, Parulekar WR, et al. Regional Nodal Irradiation in Early-Stage Breast Cancer. *N Engl J Med*. Jul 23 2015;373(4):307-16. doi:10.1056/NEJMoa1415340

330. Bruix J, Qin S, Merle P, et al. Regorafenib for patients with hepatocellular carcinoma who progressed on sorafenib treatment (RESORCE): a randomised, double-blind, placebo-controlled, phase 3 trial. *Lancet*. Jan 7 2017;389(10064):56-66. doi:10.1016/s0140-6736(16)32453-9

331. Grothey A, Van Cutsem E, Sobrero A, et al. Regorafenib monotherapy for previously treated metastatic colorectal cancer (CORRECT): an international, multicentre, randomised, placebo-controlled, phase 3 trial. *Lancet*. Jan 26 2013;381(9863):303-12. doi:10.1016/s0140-6736(12)61900-x

332. Baselga J, Zamagni C, Gómez P, et al. RESILIENCE: Phase III Randomized, Double-Blind Trial Comparing Sorafenib With Capecitabine Versus Placebo With Capecitabine in Locally Advanced or Metastatic HER2-Negative Breast Cancer. *Clin Breast Cancer*. Dec 2017;17(8):585-594.e4. doi:10.1016/j.clbc.2017.05.006

333. Wu YL, Lu S, Lu Y, et al. Results of PROFILE 1029, a Phase III Comparison of First-Line Crizotinib versus Chemotherapy in East Asian Patients with ALK-Positive Advanced Non-Small Cell Lung Cancer. *J Thorac Oncol*. Oct 2018;13(10):1539-1548. doi:10.1016/j.jtho.2018.06.012

334. Di Leo A, Jerusalem G, Petruzelka L, et al. Results of the CONFIRM phase III trial comparing fulvestrant 250 mg with fulvestrant 500 mg in postmenopausal women with estrogen receptor-positive advanced breast cancer. *J Clin Oncol*. Oct 20 2010;28(30):4594-600. doi:10.1200/jco.2010.28.8415

335. Kang YK, Ryu MH, Yoo C, et al. Resumption of imatinib to control metastatic or unresectable gastrointestinal stromal tumours after failure of imatinib and sunitinib (RIGHT): a randomised, placebo-controlled, phase 3 trial. *Lancet Oncol*. Nov 2013;14(12):1175-82. doi:10.1016/s1470-2045(13)70453-4

336. Robert NJ, Diéras V, Glaspy J, et al. RIBBON-1: randomized, double-blind, placebo-controlled, phase III trial of chemotherapy with or without bevacizumab for first-line treatment of human epidermal growth factor receptor 2-negative, locally recurrent or metastatic breast cancer. *J Clin Oncol*. Apr 1 2011;29(10):1252-60. doi:10.1200/jco.2010.28.0982

© 2024 Sherry AD et al. *JAMA Network Open.*

337. Brufsky AM, Hurvitz S, Perez E, et al. RIBBON-2: a randomized, double-blind, placebo-controlled, phase III trial evaluating the efficacy and safety of bevacizumab in combination with chemotherapy for second-line treatment of human epidermal growth factor receptor 2-negative metastatic breast cancer. *J Clin Oncol*. Nov 10 2011;29(32):4286-93. doi:10.1200/jco.2010.34.1255

338. Hortobagyi GN, Stemmer SM, Burris HA, et al. Ribociclib as First-Line Therapy for HR-Positive, Advanced Breast Cancer. *N Engl J Med*. Nov 3 2016;375(18):1738-1748. doi:10.1056/NEJMoa1609709

339. Tripathy D, Im SA, Colleoni M, et al. Ribociclib plus endocrine therapy for premenopausal women with hormone-receptor-positive, advanced breast cancer (MONALEESA-7): a randomised phase 3 trial. *Lancet Oncol*. Jul 2018;19(7):904-915. doi:10.1016/s1470-2045(18)30292-4

340. Vitolo U, Ladetto M, Boccomini C, et al. Rituximab maintenance compared with observation after brief first-line R-FND chemoimmunotherapy with rituximab consolidation in patients age older than 60 years with advanced follicular lymphoma: a phase III randomized study by the Fondazione Italiana Linfomi. *J Clin Oncol*. Sep 20 2013;31(27):3351-9. doi:10.1200/jco.2012.44.8290

341. Robak T, Dmoszynska A, Solal-Céligny P, et al. Rituximab plus fludarabine and cyclophosphamide prolongs progression-free survival compared with fludarabine and cyclophosphamide alone in previously treated chronic lymphocytic leukemia. *J Clin Oncol*. Apr 1 2010;28(10):1756-65. doi:10.1200/jco.2009.26.4556

342. Coleman RL, Oza AM, Lorusso D, et al. Rucaparib maintenance treatment for recurrent ovarian carcinoma after response to platinum therapy (ARIEL3): a randomised, double-blind, placebo-controlled, phase 3 trial. *Lancet*. Oct 28 2017;390(10106):1949-1961. doi:10.1016/s0140-6736(17)32440-6

343. Zhu AX, Rosmorduc O, Evans TR, et al. SEARCH: a phase III, randomized, double-blind, placebo-controlled trial of sorafenib plus erlotinib in patients with advanced hepatocellular carcinoma. *J Clin Oncol*. Feb 20 2015;33(6):559-66. doi:10.1200/jco.2013.53.7746

344. Carvajal RD, Piperno-Neumann S, Kapiteijn E, et al. Selumetinib in Combination With Dacarbazine in Patients With Metastatic Uveal Melanoma: A Phase III, Multicenter, Randomized Trial (SUMIT). *J Clin Oncol*. Apr 20 2018;36(12):1232-1239. doi:10.1200/jco.2017.74.1090

© 2024 Sherry AD et al. *JAMA Network Open.*

345. Krag DN, Anderson SJ, Julian TB, et al. Sentinel-lymph-node resection compared with conventional axillary-lymph-node dissection in clinically node-negative patients with breast cancer: overall survival findings from the NSABP B-32 randomised phase 3 trial. *Lancet Oncol*. Oct 2010;11(10):927-33. doi:10.1016/s1470-2045(10)70207-2

346. Perry JR, Laperriere N, O'Callaghan CJ, et al. Short-Course Radiation plus Temozolomide in Elderly Patients with Glioblastoma. *N Engl J Med*. Mar 16 2017;376(11):1027-1037. doi:10.1056/NEJMoa1611977

347. Böhle A, Leyh H, Frei C, et al. Single postoperative instillation of gemcitabine in patients with non-muscle-invasive transitional cell carcinoma of the bladder: a randomised, double-blind, placebo-controlled phase III multicentre study. *Eur Urol*. Sep 2009;56(3):495-503. doi:10.1016/j.eururo.2009.06.010

348. Kantoff PW, Higano CS, Shore ND, et al. Sipuleucel-T immunotherapy for castration-resistant prostate cancer. *N Engl J Med*. Jul 29 2010;363(5):411-22. doi:10.1056/NEJMoa1001294

349. Escudier B, Eisen T, Stadler WM, et al. Sorafenib in advanced clear-cell renal-cell carcinoma. *N Engl J Med*. Jan 11 2007;356(2):125-34. doi:10.1056/NEJMoa060655

350. Llovet JM, Ricci S, Mazzaferro V, et al. Sorafenib in advanced hepatocellular carcinoma. *N Engl J Med*. Jul 24 2008;359(4):378-90. doi:10.1056/NEJMoa0708857

351. Brose MS, Nutting CM, Jarzab B, et al. Sorafenib in radioactive iodine-refractory, locally advanced or metastatic differentiated thyroid cancer: a randomised, double-blind, phase 3 trial. *Lancet*. Jul 26 2014;384(9940):319-28. doi:10.1016/s0140-6736(14)60421-9

352. Flaherty LE, Othus M, Atkins MB, et al. Southwest Oncology Group S0008: a phase III trial of high-dose interferon Alfa-2b versus cisplatin, vinblastine, and dacarbazine, plus interleukin-2 and interferon in patients with high-risk melanoma--an intergroup study of cancer and leukemia Group B, Children's Oncology Group, Eastern Cooperative Oncology Group, and Southwest Oncology Group. *J Clin Oncol*. Nov 20 2014;32(33):3771-8. doi:10.1200/jco.2013.53.1590

353. du Bois A, Kristensen G, Ray-Coquard I, et al. Standard first-line chemotherapy with or without nintedanib for advanced ovarian cancer (AGO-OVAR 12): a randomised, double-blind, placebo-controlled phase 3 trial. *Lancet Oncol*. Jan 2016;17(1):78-89. doi:10.1016/s1470-2045(15)00366-6

© 2024 Sherry AD et al. *JAMA Network Open.*

354. Raymond E, Dahan L, Raoul JL, et al. Sunitinib malate for the treatment of pancreatic neuroendocrine tumors. *N Engl J Med*. Feb 10 2011;364(6):501-13. doi:10.1056/NEJMoa1003825

355. Scagliotti GV, Krzakowski M, Szczesna A, et al. Sunitinib plus erlotinib versus placebo plus erlotinib in patients with previously treated advanced non-small-cell lung cancer: a phase III trial. *J Clin Oncol*. Jun 10 2012;30(17):2070-8. doi:10.1200/jco.2011.39.2993

356. Motzer RJ, Hutson TE, Tomczak P, et al. Sunitinib versus interferon alfa in metastatic renal-cell carcinoma. *N Engl J Med*. Jan 11 2007;356(2):115-24. doi:10.1056/NEJMoa065044

357. Litton JK, Rugo HS, Ettl J, et al. Talazoparib in Patients with Advanced Breast Cancer and a Germline BRCA Mutation. *N Engl J Med*. Aug 23 2018;379(8):753-763. doi:10.1056/NEJMoa1802905

358. Andtbacka RH, Kaufman HL, Collichio F, et al. Talimogene Laherparepvec Improves Durable Response Rate in Patients With Advanced Melanoma. *J Clin Oncol*. Sep 1 2015;33(25):2780-8. doi:10.1200/jco.2014.58.3377

359. Butts C, Socinski MA, Mitchell PL, et al. Tecemotide (L-BLP25) versus placebo after chemoradiotherapy for stage III non-small-cell lung cancer (START): a randomised, double-blind, phase 3 trial. *Lancet Oncol*. Jan 2014;15(1):59-68. doi:10.1016/s1470-2045(13)70510-2

360. Hudes G, Carducci M, Tomczak P, et al. Temsirolimus, interferon alfa, or both for advanced renal-cell carcinoma. *N Engl J Med*. May 31 2007;356(22):2271-81. doi:10.1056/NEJMoa066838

361. Monk BJ, Herzog TJ, Kaye SB, et al. Trabectedin plus pegylated liposomal Doxorubicin in recurrent ovarian cancer. *J Clin Oncol*. Jul 1 2010;28(19):3107-14. doi:10.1200/jco.2009.25.4037

362. Piccart-Gebhart MJ, Procter M, Leyland-Jones B, et al. Trastuzumab after adjuvant chemotherapy in HER2-positive breast cancer. *N Engl J Med*. Oct 20 2005;353(16):1659-72. doi:10.1056/NEJMoa052306

© 2024 Sherry AD et al. *JAMA Network Open.*

363. von Minckwitz G, Huang CS, Mano MS, et al. Trastuzumab Emtansine for Residual Invasive HER2-Positive Breast Cancer. *N Engl J Med*. Feb 14 2019;380(7):617-628. doi:10.1056/NEJMoa1814017

364. Thuss-Patience PC, Shah MA, Ohtsu A, et al. Trastuzumab emtansine versus taxane use for previously treated HER2-positive locally advanced or metastatic gastric or gastro-oesophageal junction adenocarcinoma (GATSBY): an international randomised, open-label, adaptive, phase 2/3 study. *Lancet Oncol*. May 2017;18(5):640-653. doi:10.1016/s1470-2045(17)30111-0

365. Krop IE, Kim SB, González-Martín A, et al. Trastuzumab emtansine versus treatment of physician's choice for pretreated HER2-positive advanced breast cancer (TH3RESA): a randomised, open-label, phase 3 trial. *Lancet Oncol*. Jun 2014;15(7):689-99. doi:10.1016/s1470-2045(14)70178-0

366. Perez EA, Barrios C, Eiermann W, et al. Trastuzumab Emtansine With or Without Pertuzumab Versus Trastuzumab Plus Taxane for Human Epidermal Growth Factor Receptor 2-Positive, Advanced Breast Cancer: Primary Results From the Phase III MARIANNE Study. *J Clin Oncol*. Jan 10 2017;35(2):141-148. doi:10.1200/jco.2016.67.4887

367. Bang YJ, Van Cutsem E, Feyereislova A, et al. Trastuzumab in combination with chemotherapy versus chemotherapy alone for treatment of HER2-positive advanced gastric or gastro-oesophageal junction cancer (ToGA): a phase 3, open-label, randomised controlled trial. *Lancet*. Aug 28 2010;376(9742):687-97. doi:10.1016/s0140-6736(10)61121-x

368. Greenberg PL, Sun Z, Miller KB, et al. Treatment of myelodysplastic syndrome patients with erythropoietin with or without granulocyte colony-stimulating factor: results of a prospective randomized phase 3 trial by the Eastern Cooperative Oncology Group (E1996). *Blood*. Sep 17 2009;114(12):2393-400. doi:10.1182/blood-2009-03-211797

369. Wells SA, Jr., Robinson BG, Gagel RF, et al. Vandetanib in patients with locally advanced or metastatic medullary thyroid cancer: a randomized, double-blind phase III trial. *J Clin Oncol*. Jan 10 2012;30(2):134-41. doi:10.1200/jco.2011.35.5040

370. Herbst RS, Sun Y, Eberhardt WE, et al. Vandetanib plus docetaxel versus docetaxel as second-line treatment for patients with advanced non-small-cell lung cancer (ZODIAC): a double-blind, randomised, phase 3 trial. *Lancet Oncol*. Jul 2010;11(7):619-26. doi:10.1016/s1470-2045(10)70132-7

© 2024 Sherry AD et al. *JAMA Network Open.*

371. de Boer RH, Arrieta Ó, Yang CH, et al. Vandetanib plus pemetrexed for the second-line treatment of advanced non-small-cell lung cancer: a randomized, double-blind phase III trial. *J Clin Oncol*. Mar 10 2011;29(8):1067-74. doi:10.1200/jco.2010.29.5717

372. Seymour JF, Kipps TJ, Eichhorst B, et al. Venetoclax-Rituximab in Relapsed or Refractory Chronic Lymphocytic Leukemia. *N Engl J Med*. Mar 22 2018;378(12):1107-1120. doi:10.1056/NEJMoa1713976

373. Lin DW, Shih MC, Aronson W, et al. Veterans Affairs Cooperative Studies Program Study #553: Chemotherapy After Prostatectomy for High-risk Prostate Carcinoma: A Phase III Randomized Study. *Eur Urol*. May 2020;77(5):563-572. doi:10.1016/j.eururo.2019.12.020

374. Ravandi F, Ritchie EK, Sayar H, et al. Vosaroxin plus cytarabine versus placebo plus cytarabine in patients with first relapsed or refractory acute myeloid leukaemia (VALOR): a randomised, controlled, double-blind, multinational, phase 3 study. *Lancet Oncol*. Sep 2015;16(9):1025-1036. doi:10.1016/s1470-2045(15)00201-6

375. Socinski MA, Bondarenko I, Karaseva NA, et al. Weekly nab-paclitaxel in combination with carboplatin versus solvent-based paclitaxel plus carboplatin as first-line therapy in patients with advanced non-small-cell lung cancer: final results of a phase III trial. *J Clin Oncol*. Jun 10 2012;30(17):2055-62. doi:10.1200/jco.2011.39.5848

376. Chan JK, Brady MF, Penson RT, et al. Weekly vs. Every-3-Week Paclitaxel and Carboplatin for Ovarian Cancer. *N Engl J Med*. Feb 25 2016;374(8):738-48. doi:10.1056/NEJMoa1505067

377. Machiels JP, Subramanian S, Ruzsa A, et al. Zalutumumab plus best supportive care versus best supportive care alone in patients with recurrent or metastatic squamous-cell carcinoma of the head and neck after failure of platinum-based chemotherapy: an open-label, randomised phase 3 trial. *Lancet Oncol*. Apr 2011;12(4):333-43. doi:10.1016/s1470-2045(11)70034-1

378. Cripe LD, Uno H, Paietta EM, et al. Zosuquidar, a novel modulator of P-glycoprotein, does not improve the outcome of older patients with newly diagnosed acute myeloid leukemia: a randomized, placebo-controlled trial of the Eastern Cooperative Oncology Group 3999. *Blood*. Nov 18 2010;116(20):4077-85. doi:10.1182/blood-2010-04-277269

© 2024 Sherry AD et al. *JAMA Network Open.*
