# Supplementary material for: Differential Treatment Effects of Subgroup Analyses in Phase 3 Oncology Trials From 2004 to 2020
Source: JAMA Netw Open. 2024 Mar 28;7(3):e243379. doi: 10.1001/jamanetworkopen.2024.3379 (PMC10979321; doi:10.1001/jamanetworkopen.2024.3379)
Supplement: Supplement 2. — Data Sharing Statement [file jamanetwopen-e243379-s002.pdf]

Data Sharing Statement

Sherry. Differential Treatment Effects of Subgroup Analyses in Phase 3 Oncology Trials from 2004 to 2020. *JAMA Netw Open*. Published March 28, 2024.
doi:10.1001/jamanetworkopen.2024.3379

Data
Data available: Yes
Data types: Data (not involving human participants)
How to access data: Research data are stored in an institutional repository and will be shared upon reasonable request to the corresponding author up to 1 year following publication of the manuscript.
When available: With publication

Supporting Documents
Document types: None

Additional Information
Who can access the data: Data will be available to researchers whose proposed use of the data has been approved.
Types of analyses: For specified purpose as discussed with the corresponding author.
Mechanisms of data availability: With a signed data access agreement
Any additional restrictions: Data will be available up to 1 year after publication
